# Major Oxidative and Antioxidant Mechanisms During Heat Stress-Induced Oxidative Stress in Chickens

**DOI:** 10.3390/antiox14040471

**Published:** 2025-04-15

**Authors:** Bikash Aryal, Josephine Kwakye, Oluwatomide W. Ariyo, Ahmed F. A. Ghareeb, Marie C. Milfort, Alberta L. Fuller, Saroj Khatiwada, Romdhane Rekaya, Samuel E. Aggrey

**Affiliations:** 1 NutriGenomics Laboratory, Department of Poultry Science, The University of Georgia, Athens, GA 30602, USA or aryal.26@osu.edu (B.A.); josephine.kwakye@uga.edu (J.K.); oluwatomide.ariyo@uga.edu (O.W.A.); ahmed.ghareeb@uga.edu (A.F.A.G.); milfort@uga.edu (M.C.M.); alfuller@uga.edu (A.L.F.); 2 Department of Animal Sciences, The Ohio State University, Wooster, OH 44691, USA; khatiwada.20@osu.edu; 3 Boehringer Ingelheim Animal Health (BIAH), Gainesville, GA 30501, USA; 4 Department of Animal and Dairy Science, The University of Georgia, Athens, GA 30602, USA; rrekaya@uga.edu

**Keywords:** heat stress, chickens, reactive oxygen species, oxidative stress, antioxidant mechanisms

## Abstract

Heat stress (HS) is one of the most important stressors in chickens, and its adverse effects are primarily caused by disturbing the redox homeostasis. An increase in electron leakage from the mitochondrial electron transport chain is the major source of free radical production under HS, which triggers other enzymatic systems to generate more radicals. As a defense mechanism, cells have enzymatic and non-enzymatic antioxidant systems that work cooperatively against free radicals. The generation of free radicals, particularly the reactive oxygen species (ROS) and reactive nitrogen species (RNS), under HS condition outweighs the cellular antioxidant capacity, resulting in oxidative damage to macromolecules, including lipids, carbohydrates, proteins, and DNA. Understanding these detrimental oxidative processes and protective defense mechanisms is important in developing mitigation strategies against HS. This review summarizes the current understanding of major oxidative and antioxidant systems and their molecular mechanisms in generating or neutralizing the ROS/RNS. Importantly, this review explores the potential mechanisms that lead to the development of oxidative stress in heat-stressed chickens, highlighting their unique behavioral and physiological responses against thermal stress. Further, we summarize the major findings associated with these oxidative and antioxidant mechanisms in chickens.

## 1. Introduction

Redox homeostasis is an essential cellular mechanism present in all forms of life, responsible for the delicate balance between oxidizing and reducing reactions. Free radicals are highly reactive molecules with unpaired electrons in their outer orbital that attempt to bond with other atoms or molecules to form a stable compound [1,2]. Reactive oxygen species (ROS) and reactive nitrogen species (RNS) together form free radicals as well as other non-radical reactive substances [3]. The ROS constitute a group of molecules originating from molecular oxygen by accepting electrons and encompass both oxygen radicals, such as superoxide (O_2_^•−^), hydroxyl (HO^•^), and peroxyl (ROO^•^), and other non-radicals like hydrogen peroxide (H_2_O_2_), ozone (O_3_), and singlet oxygen (^1^O_2_) [2,4,5]. The RNS consists of nitrogen-containing radicals, including nitric oxide (^•^NO), and nitrogen dioxide (^•^NO_2_) radicals and peroxynitrite (ONOO^−^) non-radicals [6]. The ROS/RNS are produced naturally as a by-product of cellular metabolisms or from exogenous sources including heavy metals, UV rays, and air pollutants. While the low level of ROS/RNS is vital in several physiological processes, particularly the signaling pathways and immune functions, vasodilation and cardiovascular functions, tissue repair, neurotransmission, and redox regulation [7,8,9,10,11,12], excessive production of ROS/RNS depletes the body antioxidant capacity, resulting in oxidative damage of polyunsaturated fatty acids (PUFAs), proteins, carbohydrates, and DNA [13,14,15]. The adaptive mechanisms involving enzymatic and non-enzymatic antioxidants allow the cells to tolerate a certain level of oxidative stress and maintain redox homeostasis. Attempts at uplifting these adaptive mechanisms to alleviate the negative effects of oxidative injury have drawn considerable attention over the last several decades.

In animals, HS has traditionally been considered the primary cause of ROS-mediated oxidative injury [16,17,18]. With the Earth’s rising temperature, HS has become a significant challenge in animal production, particularly in tropical, subtropical, and even moderate climates, affecting a wide range of animals regardless of their natural habitat. The absence of sweat glands in birds to dissipate the heat has made them more vulnerable to HS [19,20]. In particular, HS poses a serious threat to the poultry industry, which makes a significant contribution to the United States and the global economy. Commercial broiler chickens selected for high growth rates or layers selected for high egg production are especially vulnerable to HS because of their high energy requirement, resulting in increased metabolic activity and greater heat production, which is further exacerbated by their genetic predisposition for lower heat tolerance [21,22]. Some major adverse effects of HS in chickens include decreased growth, reduced egg production, and egg quality, increased susceptibility to diseases, and mortality [23,24,25].

Given the species-specific variations and their unique physiological responses, the severity of HS and the resulting oxidative stress differs in chickens compared to other animals. However, since redox regulation is fundamental for cell survival, oxidative and antioxidant mechanisms are likely to be largely conserved in all types of cells. Unfortunately, there are very limited in vitro studies comparing these mechanisms in avian cells with those in mammalian cells. Any unique features identified in chickens are highlighted in this review. There are a handful of previous reviews that have discussed the effect of HS on production parameters as well as on the oxidative and antioxidative mechanisms. However, most of them do not address potential mechanisms and the possible cross-talks between these mechanisms during HS-induced oxidative stress. Moreover, these reviews did not specifically explain the significance of those mechanisms in chickens under HS conditions. In this article, we elaborated on major oxidative and antioxidative mechanisms, their relevance, and the previous findings under HS-induced oxidative stress, particularly focusing on chickens. Additionally, we summarized various pathways in schematic diagrams aimed at aiding the readers to comprehend these mechanisms easily and coherently. A better understanding of these molecular mechanisms will help to explore the potential nutritional interventions to elevate the cellular antioxidant mechanisms during oxidative stress.

## 2. Heat Stress-Induced Oxidative Stress—Current Understanding

**Figure 1:**
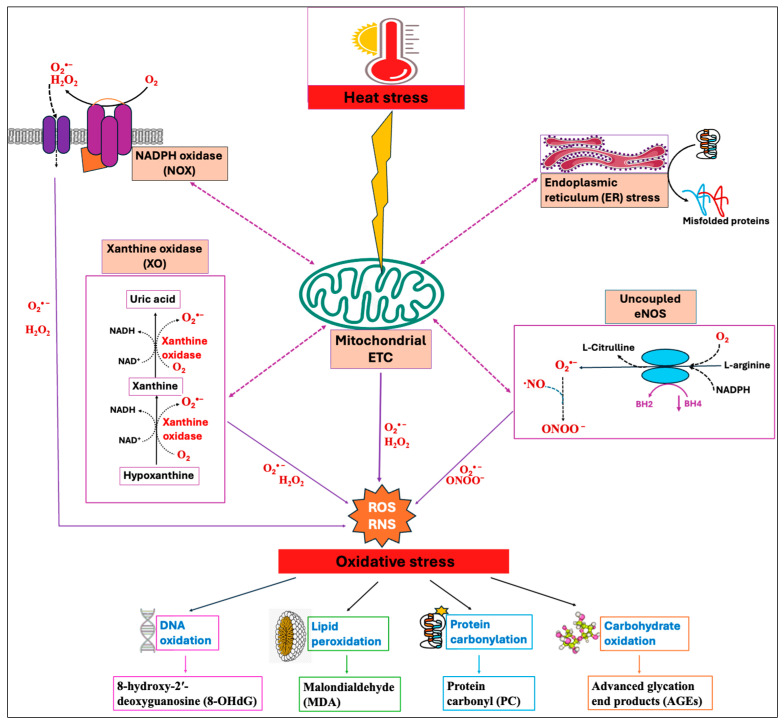
Heat stress (HS)-induced oxidative stress and the indicators of oxidative stress. Primary contributors to cellular reactive oxygen species (ROS) and reactive nitrogen species (RNS) generation include mitochondrial electron transport chain (ETC), NADPH oxidase (NOX), xanthine oxidase (XO), and uncoupled endothelial nitric oxide synthase (eNOS). Heat stress primarily triggers mitochondrial ROS production, which then activates other enzymatic systems, showing significant interplay between them. Increased levels of mitochondrial ROS disrupt the endoplasmic reticulum (ER) homeostasis, leading to the buildup of misfolded proteins, which in turn prompt mitochondria to produce more ROS. The resulting feed-forward mechanisms amplify ROS production and lead to the oxidative damage of DNA, lipids, proteins, and carbohydrates.

Among numerous enzymatic systems, four major systems, namely, NADPH oxidases (NOXs) [26], mitochondrial electron transport chain (ETC) [27], xanthine oxidase (XO) [28], and uncoupled endothelial nitric oxide synthase (eNOS) [29], have been identified as the major sources of ROS/RNS production in cells. These systems exhibit a significant interplay where the triggering of one source can activate the others, leading to feed-forward mechanisms that amplify ROS production and oxidative stress [30]. The concept of “ROS-induced ROS-release” has been proposed by many authors by which a small quantity of ROS stimulates a larger production of ROS from the mitochondria [31,32]. Heat stress has been shown to induce enzymatic systems, especially the mitochondrial electron transport chain (ETC), thereby increasing the production of ROS [33]. Moreover, HS-induced oxidative stress impairs the protein folding mechanism and promotes the buildup of unfolded and misfolded proteins, which in turn causes endoplasmic reticulum (ER) stress [34]. The resultant ER stress can further induce mitochondrial dysfunction and elevate mitochondrial ROS production [35,36], as summarized in Figure 1.

### 2.1. Mitochondrial Electron Transport Chain

During cellular metabolism, electrons transferred through the mitochondrial electron transport chain (ETC) establish a proton gradient over the mitochondrial membrane and drive ATP production. This process of ATP synthesis from ADP and Pi induced by electron transfer is called oxidative phosphorylation [37]. However, some of the electrons transferred to the ETC leak back prematurely and produce O_2_^•−^ anion radicals. Mitochondrial O_2_^•−^ radicals are mainly produced in two specific sites of the ETC, namely, complex-I [nicotinamide adenine dinucleotide dehydrogenase (NADH)] and complex-III (ubiquinone-cytochrome c reductase) [38]. Under normal metabolic conditions, complex-III is generally considered to be the most important site of ROS production [39]; however, conditions like HS can increase electron leakage and ROS production [40,41]. Mitochondrial uncoupling proteins (UCPs) are inner membrane carrier proteins that play a major role in the regulation of mitochondrial membrane potential and ROS levels through proton leakage [42,43]. This proton leakage across the mitochondrial membrane releases the energy of the proton gradient as heat and uncouples oxidative phosphorylation [44]. It has been suggested that increased ROS generation induces proton leakage, which in turn reduces ROS production [45,46].

The initial response following HS involves a rise in cellular energy requirements, doubling the energy expenditure during acute heat exposure [47]. Consequently, acute HS is shown to increase fatty acid transportation and mitochondrial β-oxidation to meet the cellular energy demand [48], thereby increasing the enzymatic activity of respiratory chain complexes [49]. However, this increase in mitochondrial energy production also leads to greater ROS generation as a result of elevated electron carrier reduction, increased membrane potential, and high mitochondrial oxygen levels [50]. Moreover, research has demonstrated that HS deactivates complex-I of the ETC and prompts its dissociation into smaller subcomplexes without impacting other complexes, thus slowing down the electron flow through ETC [33,51]. This causes an increase in ROS production due to the impairment of complex-I; however, it also induces manganese superoxide dismutase (Mn-SOD) that converts O_2_^•−^ to hydrogen peroxide (H_2_O_2_). Furthermore, the reduction of H_2_O_2_ to water is mediated by two enzymes, catalase and glutathione peroxidase. Thus, a defect in complex-I may not lead to increased ROS production if the activity of Mn-SOD is enhanced [52].

**Figure 2:**
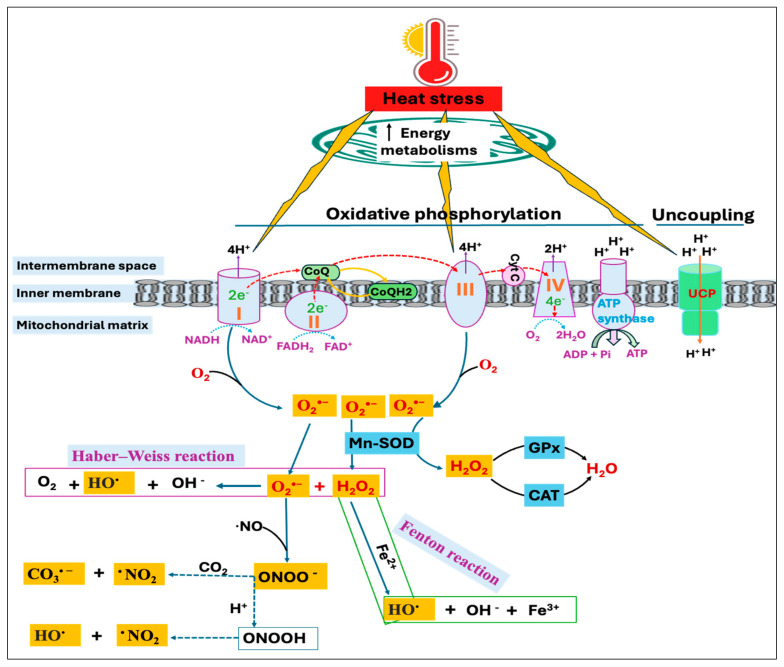
Heat stress (HS)-induced reactive oxygen species (ROS) production in mitochondria. Electrons transport through complex-I, -III, and -IV drives proton transfer to intermembrane space, creating an electrochemical proton gradient that ultimately moves protons back through ATP synthase to generate ATP. Uncoupling protein (UCP), through proton leakage, uncouples oxidative phosphorylation and regulates mitochondrial ROS production. Heat stress increases energy metabolisms, affecting the enzymatic activity of respiratory chain complexes. This alters the UCP expression as well as increases electron leakage from complex-I and complex-III, resulting in superoxide (O_2_^•−^) production, which can have several fates. Mitochondrial manganese superoxide dismutase (Mn-SOD) catalyzes the conversion of O_2_^•−^ to hydrogen peroxide (H_2_O_2_), which can be reduced to water by several cellular enzymes, including the glutathione peroxidase (GPx) and catalase (CAT). H_2_O_2_ can also result in the generation of hydroxyl radical (HO^•^) with the availability of transition metals through Fenton chemistry or by reacting with the O_2_^•−^ via Haber–Weiss reaction. Rapid inactivation of nitric oxide (^•^NO) in the presence of O_2_^•−^ resulted in the yield of peroxynitrite (ONOO^−^), a strong oxidant that subsequently undergoes protonation or decomposes into other reactive species, such as carbonate radical (CO_3_^•−^) and nitrogen dioxide radical (^•^NO_2_).

On the other hand, the O_2_^•−^ and H_2_O_2_ can react through the Haber–Weiss reaction to generate the highly reactive hydroxyl radical (HO^•^) [53] or the H_2_O_2_ can be reduced to HO^•^ in the presence of transition metals, such as iron and copper through a Fenton reaction [54]. Moreover, excessive production of O_2_^•−^ leads to the rapid inactivation of nitric oxide (^•^NO), resulting in the yield of a potent oxidant, peroxynitrite (ONOO^−^) [55]. Because nucleophilic CO_2_ is readily available, ONOO^−^ combines quickly with CO_2_ to produce carbonate radical (CO_3_^•−^) and nitrogen dioxide radical (^•^NO_2_), or it may also be reduced to form HO^•^ and ^•^NO_2_ (Figure 2). The excessive production of free radicals during HS can overwhelm the body’s antioxidant defenses, causing oxidative damage to proteins, lipids, carbohydrates, and DNA [18,56]. On the other hand, free radical-induced oxidative injury is found to be more severe and persistent in mitochondrial DNA (mtDNA) compared to nuclear DNA (nDNA) since mtDNA encodes for several proteins critical for electron transport and ROS production [57,58]. Therefore, oxidative damage to mtDNA results in a vicious cycle involving ROS generation and organelle dysregulation, ultimately triggering the apoptosis process [59].

### 2.2. NADPH Oxidase (NOX)

NADPH oxidase (NOX) proteins are a group of enzymes responsible for transferring electrons across membranes to oxygen, yielding O_2_^•−^ as the end product [60]. The extracellular O_2_^•−^ is then dismutase to H_2_O_2_, which is commonly assumed to passively diffuse back into the cells through the plasma membrane. Emerging studies suggest that aquaporin channels might serve as a preferential route for H_2_O_2_ entry into the cells [61,62]. Currently, seven known isoforms of NOX (Nox1–5, Duox1, and Duox2) have been discovered that share a common catalytic core formed by two domains: the dehydrogenase and the transmembrane domain containing the substrates and prosthetic groups that facilitate electron transfer [63]. However, these NOX isoforms are not only regulated differently and have specific cellular distributions, but also produce different types of ROS. Nox4 regulates the basal production of H_2_O_2_ [64], and Nox1 and Nox2 produce O_2_^•−^ [65], while Nox5 generates H_2_O_2_ in a Ca^2+^-dependent manner [66]. Nox2, also known as gp91phox, is the classic NADPH oxidase expressed predominantly in phagocytic cells. The study reported that in addition to mitochondria, NOX4 might also be involved in heat stress-induced ROS production and oxidative stress in avian muscle cells [67]. Other findings suggested that stimulation of mitochondrial O_2_^•−^ could elevate the activity of NADPH oxidases, resulting in increased O_2_^•−^ production in the cytosol [30,68].

### 2.3. Xanthine Oxidase (XO)

Xanthine oxidase (XO, type O), an isoform of xanthine oxidoreductase (XOR), is a key enzyme in purine metabolism catalyzing the oxidation of hypoxanthine to xanthine and xanthine to uric acid with consequent reduction of molecular oxygen [69]. This enzyme transfers electrons directly to O_2_, reducing it to generate O_2_^•−^ and H_2_O_2_ via a one-electron and a two-electron reduction, respectively [70]. Another isoform of xanthine oxidoreductase, xanthine dehydrogenase (XDH, type D), however, is not involved in ROS production [71]. Under the conditions of hyperthermia, XDH (type-D) was shown to be converted into XO (type O) in rat hepatocytes [72,73]. A limited but constant presence of XO activity was reported in the liver and kidney of chickens [74,75,76]. These observations imply that avian tissues may possess some degree of XO activity under specific circumstances; nevertheless, there is no specific study to determine if HS or hyperthermia can directly activate this enzyme in avian cells.

### 2.4. Uncoupled Nitric Oxide Synthase (NOS)

Nitric oxide synthase (NOS) is a family of oxidoreductases that catalyzes the synthesis of nitric oxide (NO) and l-citrulline, utilizing l-arginine, molecular oxygen (O_2_), and NADPH-derived electrons as substrates, while tetrahydrobiopterin (BH4) is a cofactor [77]. There are three distinct subtypes of NOS, namely, inducible NOS (iNOS), neuronal NOS (nNOS), and endothelial NOS (eNOS). The expression of eNOS and nNOS is constitutive to produce their biological effects depending on their site of localization, while iNOS is induced under stress conditions [78]. Under certain conditions such as superoxide-induced oxidation of BH4, changes in the phosphorylation state of NOS, or structural change in the functional dimer, NOS will become uncoupled and lose its ability to convert l-arginine to l-citrulline, rather than accept an electron from NADPH and transfer it to molecular oxygen to yield O_2_^•−^ instead of NO [79,80]. Thus, the production of O_2_^•−^ instead of ^•^NO leads to oxidative stress. On the other hand, ^•^NO can itself modulate oxidative stress in the cell by reacting with O_2_^•−^ to form peroxynitrite (ONOO^−^). Peroxynitrite is detrimental to the cell and exacerbates oxidative stress, even though it is less toxic compared to the O_2_^•−^ [81]. Heat stress was shown to upregulate the NOS expression, thereby elevating ^•^NO production in both in vitro and in vivo models [82,83].

### 2.5. Endoplasmic Reticulum (ER Stress)

The endoplasmic reticulum (ER) is the cellular organelle involved in the folding, modification, and synthesis of secretory and organelle-bound proteins. Protein synthesis and the subsequent folding steps are tightly regulated, and perturbation of ER homeostasis can be detrimental. Under normal circumstances, the cell maintains protein homeostasis within the ER; however, the homeostasis can be disrupted by a variety of physiological and pathological stimuli, leading to the buildup of unfolded and misfolded proteins, a condition known as ER stress [84]. Heat stress particularly elevates mitochondrial ROS production, which in turn induces oxidative stress, resulting in protein denaturation and the subsequent development of ER stress (Figure 1) [85,86]. The proteotoxic stress then activates a series of signaling pathways, known as the unfolded protein response (UPR), that aims at alleviating the stress and regaining ER homeostasis. Three ER transmembrane proteins, namely, inositol requiring 1 (IRE1), PKR-like ER kinase (PERK), and activating transcription factor 6 (ATF6), which are in inactive states during unstressed conditions, trigger UPR activation upon ER stress [87,88]. The UPR attempts to mitigate the buildup of unfolded proteins by lowering the secretory protein production, increasing molecular chaperones and protein processing enzymes as well as accelerating the degradation of misfolded or slowly folding proteins via the lysosomal pathway or ER-associated degradation (ERAD) [89,90]. However, the failure of UPR to reduce ER stress and to restore homeostasis during unresolvable ER stress conditions promotes cell death. In multiple in vitro and in vivo models, ER stress and oxidative stress are cooperative and synergistic in an amplification loop that impairs cell function and promotes pro-apoptotic signaling [34].

**Figure 3:**
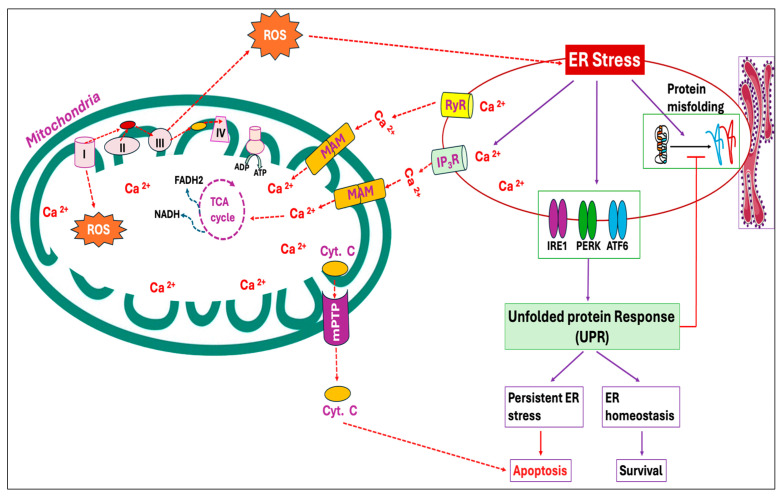
Endoplasmic reticulum (ER) stress and mitochondrial reactive oxygen species (ROS) production exhibit a positive feed-forward loop. In an attempt to mitigate the accumulation of unfolded proteins and ER stress, three ER transmembrane proteins, inositol requiring 1 (IRE1), PKR-like ER kinase (PERK), and activating transcription factor 6 (ATF6), in the ER lumen are triggered and activate a set of signaling cascades called the unfolded protein response (UPR). The ER represents the primary storage site of calcium (Ca^2+^), and under ER stress, Ca^2+^ from ER cisternae flows through the calcium release channels, inositol 1,4,5-trisphosphate receptors (IP_3_R) and ryanodine receptors (RyR). The Ca^2+^ thus released from the ER is sequestered by mitochondria through mitochondria-associated ER membranes (MAMs). Calcium overload in mitochondria triggers ROS production by increasing the respiratory chain activity and opening the mitochondrial permeability transition pore (mPTP), causing the release of cytochrome C and other pro-apoptotic factors. This, in turn, exacerbates ER stress, further releasing Ca^2+^ and optimizing mitochondrial ROS production.

Mitochondria can serve as an efficient Ca^2+^ buffer in various cell types, taking up a significant portion of cytosolic Ca^2+^ at the expense of mitochondrial membrane potential (ΔΨ_m_) [91]. The most important beneficial effect of Ca^2+^ in mitochondria is the stimulation of ATP synthesis resulting from the activation of enzymes associated with the Krebs cycle and oxidative phosphorylation [92,93]. However, several studies have reported that Ca^2+^ overload in mitochondria induces ROS production by activating the mitochondrial permeability transition characterized by the opening of the mitochondrial permeability transition pore (mPTP), a non-selective large conductance pore within the inner membrane. Opening of mPTP eventually leads to the uncoupling of oxidative phosphorylation, the release of cytochrome c and mitochondrial matrix, osmotic swelling as well as physical rupture of the mitochondrial outer membrane [94,95,96,97]. Moreover, Ca^2+^ overload has been observed to activate degradative enzymes that generate ROS capable of oxidative modifications to mitochondrial proteins and lipids [98,99,100]. During ER stress, Ca^2+^ released from the ER is obtained by mitochondria via mitochondria-associated ER membranes (MAMs) (Figure 3), resulting in mitochondrial ROS production [101,102].

### 2.6. Oxylipin Pathways

Oxylipins are oxygenated metabolites generated from the oxidation of polyunsaturated fatty acids (PUFAs), particularly arachidonic acid and eicosapentaenoic acid in animals [103]. They are produced in all organisms, and involved in cellular signaling, inflammation, and oxidative stress responses [104,105]. The enzymatic pathways of various oxylipin formation are mediated by three families of enzymes, namely, cyclooxygenase (COX), lipoxygenase (LOX), and cytochrome P450 (CYP) [106]. The specific enzyme involved determines the classes of oxylipins produced, leading to variations between different pathways. For example, the enzyme COX metabolizes arachidonic acid into prostanoids such as prostaglandins and thromboxanes, as well as hydroxy-metabolites like 11-hydroxyeicosatetraenoic acid (11-HETE) [107]. However, CYP produces HETEs, epoxyeicosatrienoic acid (EET), and dihydroxyeicosatrienoic acids (DHETs) from arachidonic acid metabolism [108]. LOX catalyzes the synthesis of mid-chain HETEs from arachidonic acid [109], and also mediates the synthesis of hydroxy fatty acids, such as leukotrienes, lipoxins, resolvins, protectins, maresins, hepoxilins, and eoxins [110,111]. These oxylipins can have either pro-inflammatory (prostaglandins, leukotrienes, HETEs) or anti-inflammatory (lipoxins, resolvins, protectins, EETs) properties. Generally, omega-3 fatty acid-derived oxylipins have anti-inflammatory functions, while omega-6 oxylipins are largely pro-inflammatory [112]. Bioactive oxylipins, particularly prostaglandins and leukotrienes, generated from the oxidation of arachidonic acid by enzymes LOX and COX, are shown to induce NOX-dependent ROS production [113,114]. Moreover, studies have demonstrated that prostaglandins induce ROS production by dissipating the mitochondrial membrane potential and redox alteration in cells [115,116]. Heat stress is shown to increase the expression of COX-2 and prostaglandin production in endothelial cells [117]. However, details on the effect of HS on the enzymatic production of oxylipins and oxylipin-mediated ROS generation in chickens remain scarce.

The non-enzymatic pathway of oxylipin production is mainly catalyzed via free radicals produced from heat, radiation, or metal ions [118]. The hydroxyl radicals (HO^•^) are the most potent initiators of lipid peroxidation, with glycolipids, phospholipids, and cholesterol being highly susceptible targets of detrimental peroxidative changes [119]. Among the various products of lipid peroxidation, hydroperoxides (LOOH) are the main primary products, while MDA and 4-hydroxynonenal (HNE) are formed as secondary products [120].

### 2.7. Cytochrome P450 Electron Transport System

Cytochrome P450s (CYPs) are hemoproteins belonging to one of the largest enzyme families, and they possess heme-thiolate as a cofactor. They transfer electrons from pyridine nucleotides (NADPH/NADH) to catalyze diverse reactions, such as hydroxylation, dealkylation, deamination, sulfoxidation, and peroxidation [121,122]. Consequently, they can act on multiple substrates, including fatty acids, steroids, drugs, organic solvents, and carcinogens, making the CYP enzymes important in xenobiotics and drug detoxification [123]. These enzymes are external monooxygenases that mediate oxidation reactions by incorporating one oxygen atom into a substrate while reducing the other oxygen atom to water [121]. Genome-wide analyses have identified 45 CYP genes in the chicken genome, revealing the distinct features of chicken CYPs, including their rapid evolution and significant divergence [124]. It has been shown that the uncoupling of CYP reactions resulted in the production of O_2_^•−^ and H_2_O_2_ [125]. Conversely, certain CYPs have been identified for their protective role against oxidative stress [126,127]. Research focusing on the effect of HS on chicken P450 enzymes is limited, particularly in understanding whether HS induces the uncoupling of P450 reactions to generate ROS or activates P450 enzymes to detoxify these ROS.

## 3. Filling the Gaps–Exploring the Potential Mechanisms

Heat stress, through the activation of enzymatic systems, results in oxidative stress. However, the exact mechanisms underlying the activation of these enzymatic systems under HS have not been fully established. Open mouth breathing (panting), increased peripheral circulation (wings flutter in birds), release of stress hormone (corticosterone/glucocorticoids), and reduced feed intake are some of the major immediate responses of animals, including the chickens, during HS. Discovering downstream mechanisms associated with these initial responses would potentially uncover the activation of enzymatic oxidant systems during HS.

### 3.1. Panting

**Figure 4:**
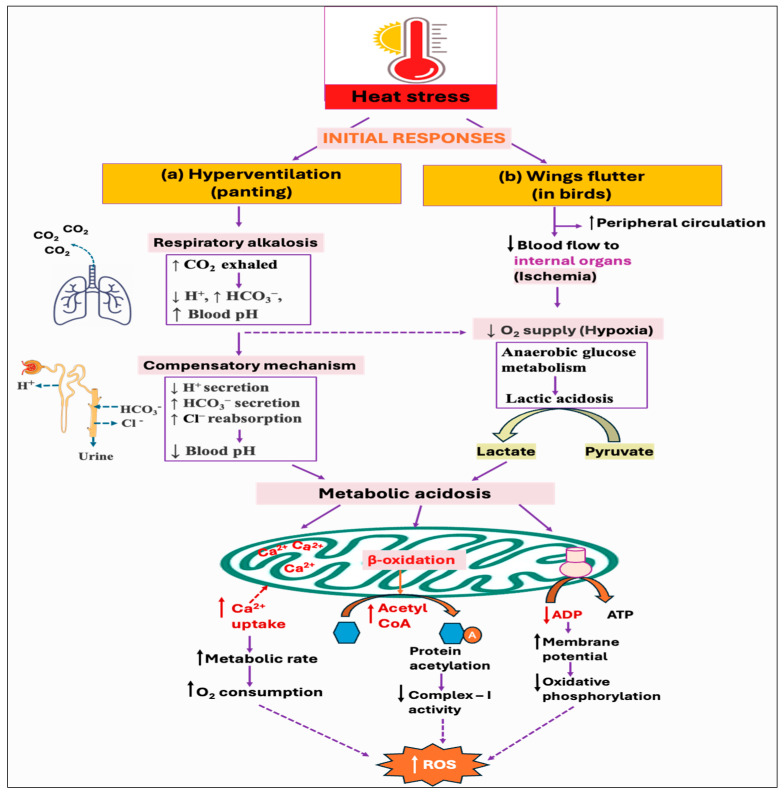
Potential downstream mechanisms associated with the initial responses, hyperventilation, and wings flutter, against heat stress: (**a**) Panting, as a way of evaporative cooling, leads to hyperventilation. Excessive loss of carbon dioxide (CO_2_) develops in respiratory alkalosis, which could eventually lead to metabolic acidosis through decreasing hydrogen ion (H^+^) secretion while increasing bicarbonate ion (HCO_3_^−^) secretion and chloride ion (Cl^−^) reabsorption from the kidney. (**b**) On the other hand, as a result of increased peripheral circulation, the blood supply to the internal organs is reduced, creating a hypoxic environment. Cells metabolize glucose anaerobically under hypoxia, resulting in the production and accumulation of lactic acid. The resultant acidosis condition stimulates mitochondria to uptake more calcium, which increases metabolic activity and subsequent reactive oxygen species (ROS) production. Acidosis can also alter oxidative phosphorylation by reducing the signal for ATP synthesis (i.e., ADP), and by modulating the mitochondrial fatty acids oxidation that decreases complex-I activity by protein acetylation. All of these mechanisms result in increased ROS generation.

Since birds lack sweat glands, panting is the major behavioral thermoregulatory response for evaporative cooling from the respiratory tract [128]. However, it results in hyperventilation and excessive loss of carbon dioxide (CO_2_), ultimately leading to respiratory alkalosis (↓ H^+^, ↑ HCO_3_^−^, ↑ blood pH) [129,130]. To counteract this imbalance and to maintain the normal blood pH, the kidneys increase the secretion of bicarbonate (HCO_3_^−^) ions, while decreasing the secretion of renal hydrogen (H^+^) ions [131]. However, this renal metabolic process develops over several days, whereas respiratory disorders have a faster impact, fluctuating CO_2_ levels in minutes to hours. As a result, acute respiratory alkalosis is associated with elevated HCO_3_^−^ levels since there has not been enough time to lower the HCO_3_^−^ levels, whereas chronic respiratory alkalosis typically involves lower-to-normal HCO_3_^−^ levels [132,133]. Some of the studies reported that hyperventilation-induced respiratory alkalosis leads to the development of compensatory metabolic acidosis [134,135]. This is because the process of HCO_3_^−^ excretion is followed by Cl^−^ retention, which drives a net increase in Cl^−^-containing acid [136]. Moreover, the fall of pCO_2_ levels (hypocapnia) under hyperventilation is found to be associated with increased lactate concentration with accompanying [HCO_3_^−^] reduction in both systemic blood and cerebrospinal fluid [137,138]. Nevertheless, the current knowledge of renal acid–base balance in chickens is limited and requires further investigation. Moreover, it remains unexplored whether respiratory alkalosis has any impact on ROS production, although metabolic acidosis is shown to exhibit both direct and indirect effects on cellular ROS production (Figure 4a).

### 3.2. Increased Peripheral Circulation (Wings Fluttering)

Heat stress leads to increased peripheral circulation to dissipate the excess heat, thus reducing the blood flow (ischemia) to internal organs, including the liver, kidney, and gastrointestinal (GI) tract [139]. Alternatively, thermal panting-induced hypocapnia and alkalosis might elevate oxygen demands due to the added workload on the respiratory muscles [140,141], and shift the oxyhemoglobin dissociation curve to the left, reducing oxygen delivery to perfused tissues [142,143]. The resulting hypoxic condition (lack of oxygen) induces the cells to metabolize glucose anaerobically and produce lactate, thus resulting in lactic acidosis [144,145]. However, there is a contrasting finding suggesting that the blood flow distribution in chicken remained unchanged in most organs during hyperventilation-induced hypocapnic alkalosis [146].

Some studies have provided various insights into how metabolic acidosis exerts substantial stress on mitochondria. Acidosis is shown to inhibit oxidative phosphorylation in skeletal muscle and may decrease the mitochondrial oxidative capacity [147,148,149]. During acidosis, there is a reduction in the specific signal for oxidative phosphorylation (i.e., [ADP]) because of the pH-induced shift in creatine kinase (CK) equilibrium, resulting in a lower signal for ATP supply from mitochondria [150,151]. The physiological rate of mitochondrial ROS production is inversely associated with the cytosolic ADP availability [152]. Thus, a reduction in the ADP levels leads to a rise in mitochondrial membrane potential that in turn decreases the respiratory rate and stimulates ROS production due to the highly reduced state of the electron transport chain components. Moreover, mitochondria increase Ca^2+^ uptake in response to acid load and to counteract high cytosolic Ca^2+^ influx, which, in turn, stimulates ROS generation from the respiratory chain [153]. It has been suggested that Ca^2+^ induces ATP synthesis through the activation of enzymes involved in the mitochondrial Krebs cycle and oxidative phosphorylation. This increase in metabolic rate consumes more oxygen, thereby increasing electron leakage and free radical generation from the electron transport chain components [154]. Furthermore, Ca^2+^ also changes the three-dimensional conformation of the electron transport chain complexes, inducing mitochondrial ROS production. Moreover, acidosis was found to modulate fatty acid metabolism in cells and decrease the respiratory chain complex-I activity through hyperacetylation of proteins [155], as summarized in Figure 4b.

### 3.3. Stress Hormone

Stressors, including the HS, have been identified to activate the hypothalamus–pituitary–adrenal (HPA) axis that is accountable for the secretion and regulation of glucocorticoid (GC) hormone, also called cortisol or corticosterone, from the adrenal gland into the bloodstream [156,157]. Glucocorticoids (GCs) are well known to act through the glucocorticoid receptor (GR, NR3C1). When not bound to a ligand, the GR is mainly located in the cytosol as part of a large heterocomplex that includes chaperon proteins (HSP90, HSP70, p23) and immunophilins (FKBP51 and FKBP52) [90,158]. The binding of GC induces a conformational change in the GR, leading its associated proteins to dissociate. Subsequently, the GR translocates quickly into the nucleus, where it directly binds to glucocorticoid response elements (GREs) and mediates target gene expression, a process termed trans-activation [159]. Moreover, the GR can also modulate the target gene’s transcription by binding with other transcription factors, such as the STAT family, AP1, and NF-κB, thereby inhibiting them from interaction with their binding site on DNA, a process called trans-repression [160]. In addition, GRs are also found in mitochondria, so they can directly mediate the effect of GC on mitochondria by transcribing the genes encoded by mitochondrial DNA (mtDNA) [161,162]. It has been shown that GCs exert a biphasic response in regulating mitochondrial metabolic activities. The enzymatic activity of specific respiratory chain complex subunits and mitochondrial biogenesis was shown to be stimulated by short-term exposure to stress-level GCs, while extended GC exposure led to mitochondrial structural alterations, respiratory chain dysfunction, excessive ROS production, apoptosis, and eventual cell death [163,164]. Harmful impacts of GCs on mitochondria are exerted through downregulating the binding of GR with the cytoprotective protein, B-cell lymphoma 2 (BCL2), which has been known to prevent the formation of Bax-containing pores on the outer membrane of mitochondria, thus decreasing the leakage of calcium and cytochrome C from mitochondria [165,166]. Additionally, GCs have been found to suppress the activities of cytochrome c oxidase as well as complex-I and -V [167,168]. Beyond these mechanisms, binding of GC with the GR in the cell membrane exerts several rapid, nongenomic effects through alterations in kinase activity, including phosphatidylinositol 3-kinase (PI_3_K), protein kinase B (AKT), and MAP kinases (MAPKs) [160].

**Figure 5:**
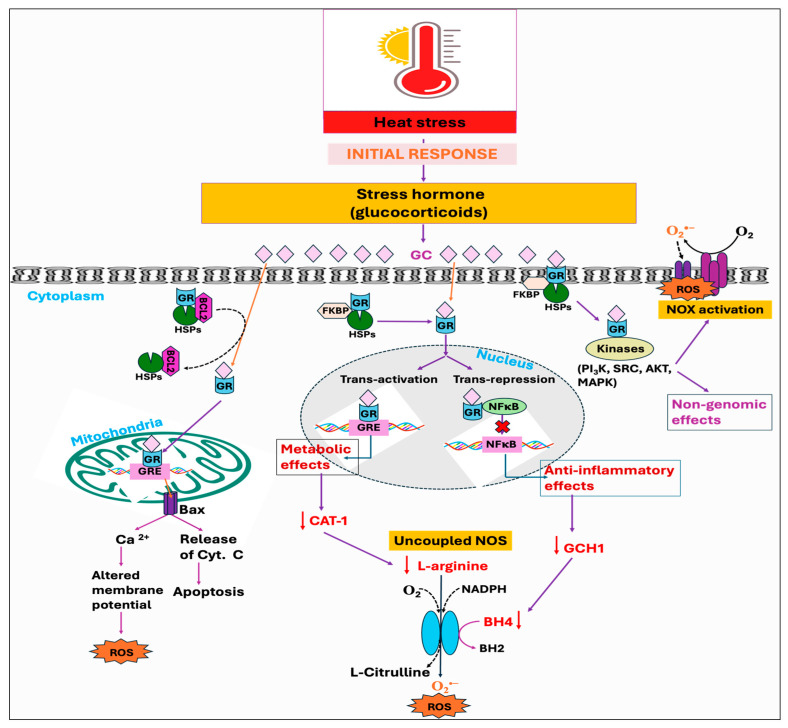
Potential downstream mechanisms associated with the initial response, stress hormone, against heat stress. The conventional and well-established mode of action of glucocorticoid (GC) is through binding to its receptor, glucocorticoid receptors (GRs). Upon nuclear localization and binding to its genomic response element (GRE), the GC-GR complex exhibits various anti-inflammatory and metabolic effects, including the deficiency of GTP cyclohydrolase I (GCH1) and downregulation of cationic amino acid transporter 1 (CAT-1). This, in turn, decreases the bioavailability of tetrahydrobiopterin (BH4) and L-arginine respectively, leading to nitric oxide synthase (NOS) uncoupling. Moreover, GR associated with heat shock protein (HSP) in mitochondria forms a complex with the anti-apoptotic protein B-cell lymphoma 2 (BCL2). Chronic exposure to the GC downregulates the binding of GR with the Bcl-2, which leads to the formation of Bax pores and subsequent leakage of calcium ions and cytochrome c. The GC also exerts its rapid non-genomic effects, such as NADPH oxidase (NOX) activation, via modulation of various kinases. Therefore, the combined effects of mitochondrial dysfunction, NOS uncoupling, and NOX activation ultimately lead to a rise in reactive oxygen species (ROS) production.

Studies have further demonstrated that GC enhances ROS generation through the activation of the NOX enzyme, and by inhibiting coupling to ATP formation during oxidative phosphorylation [169]. The GC-GR-induced ROS production is presumably mediated by src/syk/PI_3_K/AKT signaling pathways through NOX activation [169]. Glucocorticoids have also been reported to induce ROS production by limiting BH4 availability through the inhibition of inflammatory mediators, namely, GTP cyclohydrolase I (GCH1), the rate-limiting enzyme for de novo BH4 synthesis [170]. This limitation, in conjunction with the decreased availability of L-arginine due to the inhibition of cytokines affecting the CAT-1 arginine uptake transporter, leads to NOS uncoupling [170,171]. These effects are attributed to the anti-inflammatory properties of GCs, mediated either by genomic interaction of GR and NF-kB or through non-genomic anti-inflammatory pathways (Figure 5). Another mechanism whereby GCs exert a deleterious effect was found to be initiated by extracellular glucose. The stress-elicited rise in GCs subsequently increases blood glucose levels through gluconeogenesis, which has indirectly been demonstrated to decrease mitochondrial motility [167].

### 3.4. Reduced Feed Intake

Reduced feed intake during HS is the adaptive strategy to minimize the generation of internal heat by decreasing metabolic activities in animals [172,173]. This is because HS increases the secretion of leptin and adiponectin, along with their receptor expression [174,175], while leptin stimulates the hypothalamic axis and reduces feed intake [176]. The energy deficit condition activates glucagon secretion from pancreatic alpha cells, resulting in lipolysis and protein breakdown in muscle [177,178,179]. Importantly, glucagon has been shown to enhance plasma lipoprotein lipase-like activity both in vivo and in vitro [180]. Moreover, the lipoprotein lipase activity is found to be elevated in plasma, muscle, and heart under stressful condition [181], thereby increasing the breakdown of triglycerides (TG) into free fatty acids (FFAs) and glycerol, and FFAs are then utilized by peripheral tissues as an energy source [182]. The elevated concentration of FFAs in circulation also increases their uptake, re-esterification into TG, and subsequent storage in adipose tissues, resulting in greater fat deposition in HS birds [183,184]. Conversely, an increased amount of free fatty acids (FFAs), particularly polyunsaturated fatty acids (PUFAs), are shown to activate the NOX-dependent ROS generation in neutrophils [185,186]. This is because the enzymatic oxidation of PUFAs produces bioactive oxylipins, such as leukotriene B4 (LTB4), which triggers NOX activation [187,188].

In addition, oxidation of FFAs in mitochondria interrupts the electron transport from complexes I and III, thereby augmenting the ROS production [189,190,191]. Because glycolysis is the central metabolic pathway, the respiratory chain is optimized for glucose oxidation, exporting cytoplasmic NADH to the mitochondria via the malate/aspartate shuttle. This means that for every two electrons that enter from FADH_2_, ten electrons enter through NADH. This default chain is therefore optimal at a FADH_2_:NADH ratio of 0.2. Using long-chain fatty acids (LCFAs), such as palmitic acid (16 carbons), results in an FADH_2_:NADH ratio of ~0.48 [192]. Therefore, this increased FADH_2_:NADH ratio resulting from the oxidation of saturated FAs in mitochondria, in turn, decreases the CoQ to CoQH_2_ ratio (a high CoQH_2_:CoQ ratio), which reduces the concentration of electron acceptors for complex-I, leading to the formation of free radicals [192]. In addition, an increased CoQH_2_:CoQ ratio promotes reverse electron transfer (RET)-induced mitochondrial ROS along with the elevated membrane potential [193]. The resulting ROS production from the oxidation of FFAs was demonstrated to mobilize and release the cytochrome c from mitochondria via an unknown mechanism, thereby disturbing electron transport from complex-III to complex-IV [194,195]. Moreover, palmitate-induced ROS formation impairs ER redox status and affects protein folding machinery and augments misfolded or unfolded proteins, leading to ER stress [34,196]. This ER stress can exacerbate mitochondrial dysfunction, further stimulating the generation of mitochondrial ROS [35,36]. Conversely, unsaturated fatty acids are shown to prevent ROS generation, thereby antagonizing the detrimental effects of saturated fatty acid oxidation [197,198].

**Figure 6:**
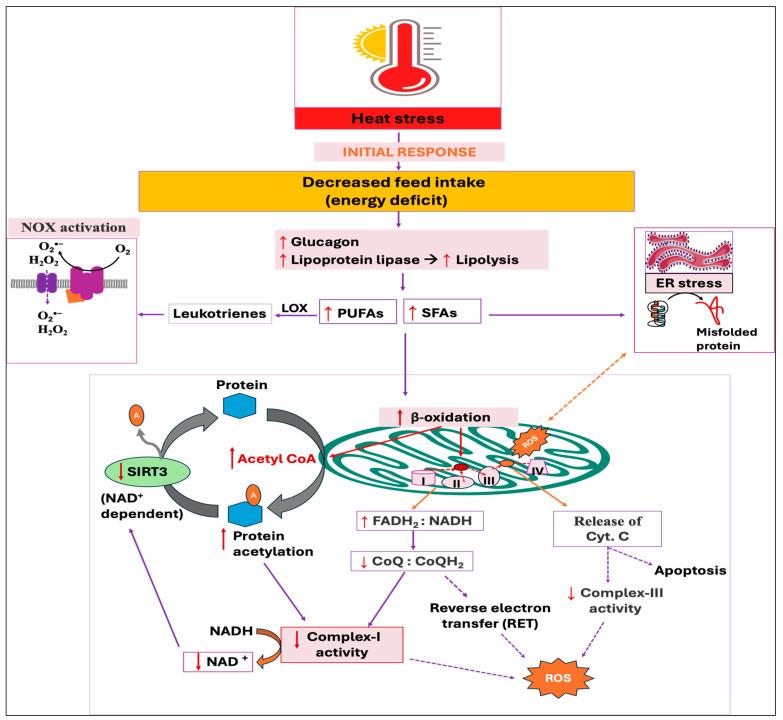
Potential downstream mechanisms associated with the initial response, decreased feed intake, against heat stress. Energy deficit condition activates lipolysis and subsequent release of free fatty acids (FFAs), with polyunsaturated fatty acids (PUFAs) activating NADPH oxidase (NOX) and saturated fatty acids (SFAs) triggering endoplasmic reticulum (ER) stress. The subsequent beta-oxidation of FFAs, especially the SFAs, increases the flavin adenine dinucleotide and reduced nicotinamide adenine dinucleotide ratio (FADH_2_:NADH), which reduces the number of electron acceptors for complex-I, ultimately increasing electron leakage and reactive oxygen species (ROS) production. Excessive acetyl-CoA generated from beta-oxidation increases the acetylation of mitochondrial proteins via both enzymatic and non-enzymatic mechanisms, which further reduces complex-I activity. The decreased NAD^+^:NADH ratio resulting from complex-I defect suppresses the deacetylase sirtuin 3 (SIRT3) enzyme, aggravating protein acetylation and ROS production. The cytochrome-C that transfers electrons from complex-III to -IV is released from the mitochondria as a result of ROS accumulation, finally inducing apoptosis.

In addition, it has been revealed that fatty acid oxidation enhances the acetylation of mitochondrial proteins, which are enzymes involved in energy metabolism [199]. There is direct evidence that indicated acetyl-CoA produced through β-oxidation of fatty acid drives mitochondrial protein acetylation [199,200] by both enzymatic (acetyltransferase-catalyzed acetylation) [201] and non-enzymatic [202,203] mechanisms. This increased acetylation of mitochondrial proteins reduces the activities of the ETC, which in turn inhibits the oxidation of NADH and a subsequent rise in ROS production [204]. Moreover, the resulting complex-I defect causes the NAD^+^:NADH ratio to decrease, suppressing the NAD^+^-dependent deacetylase sirtuin 3 (SIRT3) (Figure 6). SIRT3 exhibits potent deacetylase activity in mitochondria and overturns the inhibitory effects of acetylation, thereby enhancing oxidative metabolism along with the accompanying stimulation of ROS-mitigating systems, including SOD2 [205,206]. From the above-mentioned findings, it is promising that FFA oxidation increases ROS production; however, there are contrary findings indicating that an increase in β-oxidation alleviates oxidative stress, whereas limiting the mitochondrial fatty acid uptake intensifies ROS production [207,208].

## 4. Indicators of Oxidative Stress

Elevated ROS production usually results in the oxidation of DNA, proteins, carbohydrates, and lipids, leading to a bioenergetic failure [209]. Biomarkers such as protein carbonyl (PC), malondialdehyde (MDA), 8-hydroxy-2′-deoxyguanosine (8-OHdG), and advanced glycation end product (AGEs) are used to assess the degree of protein, lipid, DNA, and carbohydrate oxidation, respectively [13,15].

### 4.1. Lipids

The primary phospholipid constituents of mitochondrial membranes are abundant in unsaturated fatty acids, making them vulnerable to free radical injury through the peroxidation of double bonds [210]. The major product of polyunsaturated fatty acid (PUFA) peroxidation is MDA, and HS is shown to increase both the mitochondrial and plasmatic MDA levels significantly [211]. This increased level of MDA then reacts with DNA bases, leading to gene mutation [212], whereas the other product of lipid peroxidation, 4-hydroxy-2-nonenal (HNE), reacts with various amino acids such as cysteine (Cys), histidine, and lysine residues, thus affecting the functions of the enzyme [213].

### 4.2. Proteins

ROS can oxidize amino acids (AAs), especially sulfur-containing AAs, protein backbone, and cross-link the proteins [214]. Protein oxidation can be either reversible or irreversible. It primarily involves the oxidation of cysteine thiols (-SH) into cysteine sulfenic acid (R-SOH), which then may react with nearby thiols to form disulfide bonds (S-S) or be irreversibly converted to sulfinic (R-SO_2_H) and sulfonic acids (R-SO_3_H) [215,216]. Reversible protein oxidation involves glutathionylation, nitrosylation, sulfenation, and disulfide formation of cysteine residue, while irreversible protein oxidation includes carbonylation of arginine, lysine, proline, or threonine residues and nitration of tyrosine residue [217,218]. Moreover, ROS may oxidize mitochondrial proteins directly to form disulfide bonds between cysteine residues, methionine sulphoxide from methionine residues, and carbonyl groups in the side chains of serine, arginine, lysine, proline, histidine, and threonine residues [219,220]. Aggregation and accumulation of these oxidized protein derivatives can eventually cause cell death [221]. Because protein oxidation results in the formation of carbonyl groups, PCs are easily detectable and have been employed as a quantitative indicator of protein oxidation and oxidative stress [222].

### 4.3. Carbohydrates

Glycation or non-enzymatic glycosylation is the process where a reducing sugar covalently attaches with amino groups in proteins, lipids, or DNA macromolecules through the Maillard reaction, leading to the formation of AGEs [223]. Although AGEs are continuously produced as a normal by-product of metabolic processes, their formation was found to be elevated under conditions of hyperglycemia and oxidative stress [224,225]. In turn, AGEs bind with their receptor RAGE, activate pro-inflammatory and pro-oxidant pathways, and further amplify oxidative stress, generating a synergistic feed-forward loop that advances their pathophysiological functions [226,227]. Moreover, aggregation of AGEs has been associated with protein misfolding, ER stress, mitochondrial dysfunction, and cellular apoptosis [228,229].

### 4.4. DNA

Free radical-mediated DNA oxidation can alter purine and pyrimidine bases [230], or result in double-strand DNA breaks by directly damaging the sugar backbone with high-energy impact [231]. Moreover, the accumulation of ROS can damage not only nuclear DNA, it also lead to mitochondrial DNA lesions, strand breaks, and degradation [232]. The primary products resulting from DNA base damage include thymine glycol among pyrimidines [233] and 8-OHdG among purines [234]. The enzymatic repair systems can excise the 8-OHdG residues from the DNA, allowing them to circulate in the blood and be excreted in the urine [235].

### 4.5. Findings in Chickens

There are extensive studies on HS-induced oxidative stress in chickens, focusing particularly on the mRNA expression and enzymatic activity of various genes, yet the knowledge regarding mitochondrial ROS production in avian cells remains limited. Avian mitochondria were found to generate significantly less ROS than mammals, with rat mitochondria producing nearly four times more hydrogen peroxide than pigeon mitochondria when using pyruvate/malate as substrates [236]. This lower ROS production has been related to the greater longevity of the birds as compared to mammals of the same body mass [237]; however, no difference in mitochondrial ROS production was observed between long-living parrots and short-living quails [238]. Moreover, liver mitochondrial membranes in pigeons exhibited more resistance to lipid peroxidation compared to rats because of the lower level of fatty acid unsaturation in the pigeon’s membrane [239]. When exposed to pro-oxidants like 95% oxygen, H_2_O_2_, and paraquat, avian cells from long-lived bird species demonstrated greater resistance to oxidative stress and DNA damage compared to mouse cells [240]. Notably, there are no supporting data directly comparing ROS production between commercial chickens and mammals. Commercial broilers or layers, which are genetically selected for their rapid growth and higher production, may exhibit distinct oxidative stress dynamics compared to their wild-type counterparts.

In the skeletal muscle mitochondria of heat-stressed broilers, there was a slight increase (2.7%) in mitochondrial membrane potential, leading to a significant rise (47%) in ROS production [241]. However, the increase in mitochondrial membrane potential and the subsequent rise in ROS production persisted only until day 9 of HS, suggesting that chickens may adapt to HS over time [242]. The avian uncoupling protein (*AvUCP*) that reduces ROS levels by generating a proton leak was downregulated in the muscle cells post-HS [243]. However, another study has found the upregulation of *AvUCP* and downregulation of *NOX4* mRNA expressions during HS [244]. The variation in results among separate studies might be attributed to different experimental designs and differences in the duration of HS.

Heat stress was found to significantly increase the H_2_O_2_ and PC concentration in the liver of broilers while the MDA and 8-OHdG levels were not changed [245]. However, other studies identified that HS led to a significant increase in both the 8-OHdG content and AGE level in the chicken liver, suggesting severe oxidative damage to DNA and carbohydrates [14]. Moreover, HS significantly increased the levels of muscle carbonyl (2.39 vs. 1.91 nmol/mg), AGE (1.91 vs. 1.6 ng/mg), and MDA (222.9 vs. 181.63 nmol/g) compared to the control group [246]. Similarly, broilers exposed to chronic HS exhibited a higher MDA level and increased concentration of ROS with reduced concentration of ATP in the liver [247]. They further identified that chronic HS enhanced the mRNA expression of ER transmembrane proteins and UPR signaling components, such as *PERK*, *ATF4*, *IRE1*, and eukaryotic translation initiation factor 2 alpha (*eIF2α*). However, the mRNA expression of *PERK* and *eIF2α* in the *P. major* showed no significant differences between the HS and control groups, while the expression of *ATF4* was significantly higher in the HS group [248]. Acute HS reduced the total nitric oxide synthase (tNOS) activity while elevating the plasma NO levels in broilers [249]. There was an increase in blood glucose levels in HS chickens compared to the control group, likely due to a stress-induced rise in glucocorticoids [250]. Finally, hyperventilation-induced respiratory alkalosis under HS conditions has been shown to reduce the activity of carbonic anhydrase, which is a critical enzyme in forming the eggshell [251]. As a result, lower concentrations of calcium and carbonate are secreted by the shell gland, resulting in thin and weak eggshells [252,253].

## 5. The Antioxidant Defense Mechanisms

**Figure 7:**
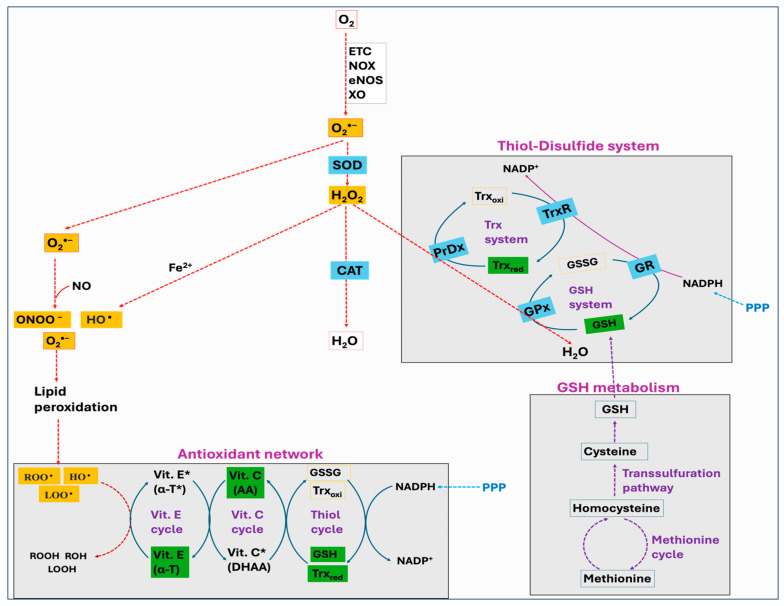
Schematic representation of major enzymatic and non-enzymatic antioxidant defense systems. Superoxide dismutase (SOD) is the forefront antioxidant that neutralizes superoxide (O_2_^•−^) to hydrogen peroxide (H_2_O_2_), which is then detoxified to water by catalase (CAT) and the thiol–disulfide system, involving glutathione peroxidase (GPx) and peroxiredoxin (PrDx). The antioxidant network refers to the synergistic relationship between different antioxidants such as vitamin E, vitamin C, and glutathione (GSH), where one replenishes the original properties of another. Vitamin E scavenges lipid peroxyl radicals (LOO^•^), peroxyl radicals (ROO^•^), and hydroxyl radicals (HO^•^) via a chain breaking mechanism and protects the cellular membranes. Cellular GSH, synthesized endogenously from methionine and cysteine, and cellular thioredoxin (Trx) are the important components of the thiol–disulfide system and the antioxidant network. Nicotinamide adenine dinucleotide phosphate (NADPH) serves as a reducing agent by donating electrons to oxidized Trx via thioredoxin reductase (TrxR), or to oxidized glutathione (GSSG) via glutathione reductase (GR).

Cells have both enzymatic and non-enzymatic antioxidant systems that function cooperatively to protect cells from oxidative injury. Antioxidants exert their effects via various mechanisms: the chain-breaking mechanism in which an electron is donated to the free radical, thereby stabilizing it and preventing the continuation of the reaction; a quenching mechanism involving deactivation of the catalyst that initiates radical formation; a co-antioxidant action; or regulation of gene expression [254,255]. The major enzymatic antioxidants consist of superoxide dismutase (SOD), catalase (CAT), glutathione peroxidase (GPx), and peroxiredoxin (PrDx), whereas the non-enzymatic antioxidants comprise glutathione (GSH), thioredoxin (Trx), lipoic acid, vitamins C and E, carotenoids, melatonin, flavonoids, and other compounds. Many authors have regarded the enzymatic antioxidants, namely, SOD, CAT, and GPx, as a first line of antioxidant defense system because of their prime importance in the overall antioxidant defense strategy, especially about O_2_^•−^. However, PrDx represents another important enzyme system, which in addition to SOD, CAT, and GPx, performs a crucial role in protecting cells against oxidative stress. Furthermore, the function of GPx and PrDx is exclusively dependent on non-enzymatic antioxidants such as GSH and Trx, and there exists a significant interplay between the enzymatic and non-enzymatic antioxidant systems (Figure 7). In this Section, we discuss various mechanisms associated with the enzymatic and non-enzymatic antioxidant systems.

### 5.1. Superoxide Dismutase (SOD)

The SODs represent the primary and most crucial antioxidant enzymes in defending against ROS, especially O_2_^•−^ radicals. They catalyze the breakdown of O_2_^•−^ into H_2_O_2_, which is then further detoxified by the complementary actions of CAT and GPx [256]. Depending on the metal cofactor and cellular localization, three major types of SODs are identified, which include copper–zinc SOD (Cu/Zn-SOD or SOD1) located primarily in cytoplasm or secreted into the extracellular fluid, manganese-containing SOD (Mn-SOD or SOD2) located in the mitochondrial matrix, and an extracellular SOD (EC-SOD or SOD3) [257].

### 5.2. Catalase (CAT), Glutathione Peroxidase (GPx), and Peroxiredoxin (PrDx)

Three major antioxidant enzymes, namely, CAT, GPx, and PrDx, are primarily responsible for intracellular peroxide metabolism. The CAT is an iron-containing peroxidase, found primarily in peroxisomes, that catalyzes H_2_O_2_ into H_2_O and O_2_. However, CAT is activated only when the concentration of H_2_O_2_ is significantly higher than physiological thresholds, and it is expressed much lower compared to GPx and PrDx [258]. While CAT operates independently of reducing agents and catalyzes H_2_O_2_ exclusively, GPx and PrDx are dependent on intracellular electron donors to perform their peroxidase activity targeting both H_2_O_2_ and organic peroxides. Antioxidant GPx is a selenium-containing peroxidases family, and unlike CAT, GPx senses a small increase in H_2_O_2_ concentration [258]. The PrDx, distinct from heme-dependent CAT and the selenium-dependent GPx, does not require cofactors. For a long time, the role of PrDx was overshadowed by the well-studied oxidative stress defense enzymes CAT and GPx, which are considered as the major defender of cells against hydroperoxides. However, PrDxs have now been identified as ubiquitous enzymes in numerous organisms, involved in the neutralization of H_2_O_2_, alkyl hydroperoxides (ROOH), and ONOO^−^ [259,260,261]. The GPx and PrDx are the components of the cellular thiol–disulfide redox system, the details of which will be discussed herein.

### 5.3. Thiol–Disulfide Redox System

**Figure 8:**
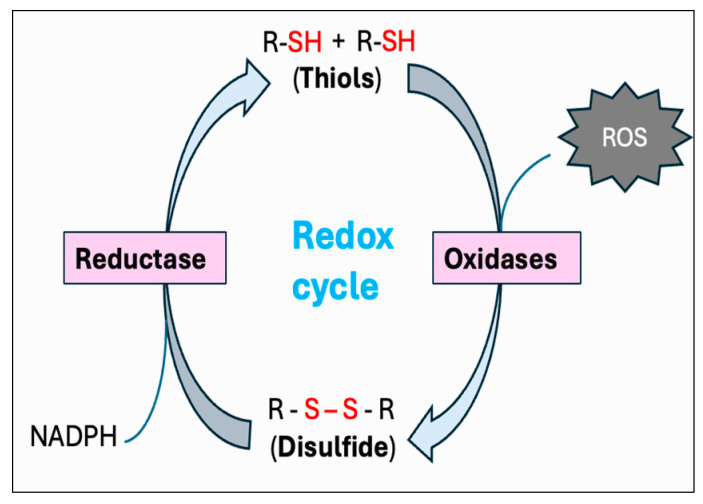
Thiol–disulfide-mediated redox cycle. By donating electrons to reactive oxygen species (ROS), thiols (R-SH) function as reducing agents to neutralize ROS into less toxic byproducts, while they become oxidized to form disulfides (R-S-S-R). Conversion of thiols to disulfide is mediated by a variety of protein disulfide isomerase (PDI) oxidases, including glutathione peroxidase (GPx) and peroxiredoxin (PrDx). Specific reductase then restores disulfides to reduced thiols by utilizing the cellular reducing agents, including NADPH. This interconversion between thiols and disulfide represents the redox cycle.

**Figure 9:**
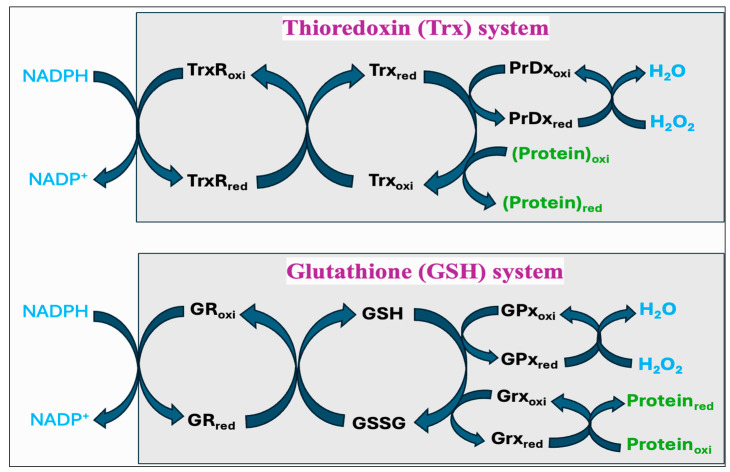
The thioredoxin (Trx) system and glutathione (GSH) systems in regulating the thiol–disulfide-mediated redox state of the cell. Both systems utilize NADPH as a final electron donor; however, they operate via different components in reducing the proteins and neutralizing hydrogen peroxide (H_2_O_2_) to water. The Trx system includes thioredoxin reductase (TrxR), thioredoxin (Trx), and peroxiredoxin (PrDx) while the GSH system comprises glutathione reductase (GR), glutathione (GSH and GSSG), and glutathione peroxidase (GPx) or glutaredoxin (Grx).

Thiols are organosulfur compounds containing a constituent sulfhydryl (R-SH) group, which renders thiols highly prone to oxidation. They are present in all biological systems, and some of the physiologically significant thiols consist of amino acids such as cysteine (Cys) and homocysteine (Hcy), coenzyme A, and dihydrolipoic acid [262]. Thiols can function as reducing agents by donating electrons to ROS, thereby neutralizing ROS into less toxic byproducts while they become oxidized to form disulfides (R-S-S-R). Structural disulfide bond formation in proteins is catalyzed by different types of oxidoreductases that encompass several protein disulfide isomerase (PDI) oxidases, including PrDx and GPx [263,264,265]. In biological systems, particular reductases restore disulfides to reduced thiols by using cellular reducing agents, including NADPH or NADH. The switch between thiols (dithiol) and disulfide groups represents a redox reaction, with a reduced state of free dithiol and the oxidized state of disulfide form (Figure 8). This reaction aids in the maintenance of a balanced oxidoreductive or redox environment inside the cell [266]. The Trx and GSH systems primarily regulate the thiol–disulfide-mediated redox status of the cell [267,268]. Although these systems operate via different mechanisms, they both utilize NADPH, mainly derived from the pentose phosphate pathway (PPP), as a reducing agent (Figure 9).

The Trx system is composed of NADPH, thioredoxin reductase (TrxR), Trx, and PrDx. Reduced Trxs (Trx_red_) are potent reductases with broad substrate specificity that efficiently reduce disulfide bonds into thiols [269]. One of the important targets of Trx_red_ is the antioxidant enzyme PrDx, which in turn mediates the reduction of peroxides through receiving electrons from Trx_red_. Moreover, Trxs are the main protein disulfide reductases in the cell to reduce and activate the proteins, while they become oxidized. The oxidized Trx (Trx_oxi_) is restored back to its reduced state (Trx_red_) by an enzyme TrxR, using NADPH as a final electron donor [270]. In mitochondria, it is reported that peroxiredoxin-3 (PrDx-3) scavenges 90% of H_2_O_2_, indicating the Trx system as the main pathway to eliminate mitochondrial H_2_O_2_ [271].

The GSH system consists of NADPH, glutathione reductase (GR), GSH, and GPx or glutaredoxin (Grx). The GSH system can protect the cells from the detrimental effects of oxidative stress through several pathways. GSH can reduce disulfide bonds in proteins through a Grx-involved reaction. This is crucial to restore the activity of several important proteins and pathways following the alterations in the cellular redox state [272,273,274]. Grx exists in two primary forms, one localized in the cytosol (Grx1) and the other present in the mitochondria and nuclei of mammalian cells (Grx2) [275]. While Grx1 obtains electrons from GSH, Grx2 can accept electrons from both GSH and TrxR2 [276]. The GSH-Grx system can therefore function as an alternative system to decrease the levels of Trx when the electrons flow from TrxR1 is inhibited [277]. Moreover, GSH transfers an electron to GPx that alleviates oxidative stress by neutralizing different types of ROS [267,278]. The GR provides electrons to GSSG by utilizing NADPH as the final electron donor, reconverting GSSG back to GSH to maintain a normal GSH:GSSG ratio [279].

### 5.4. Significance of Thiol–Disulfide Redox Status

The cellular thiol–disulfide redox balance is shaped by the distributions of thiols and disulfides across various subcellular compartments. The reduced forms of Trx and GSH serve as vital cellular thiol reservoirs, primarily associated with the neutralization of ROS. Reduction in the GSH:GSSG or Trx_red_:Trx_oxi_ ratios is often linked to the detrimental impacts of oxidative species, eliciting death or survival signals [280]. Consequently, the thiol:disulfide ratio serves as an important indicator of oxidative stress, since maintaining reduced states of GSH and Trx is critical for numerous cellular processes, including the stability and activity of various proteins such as enzymes GPx and PrDx, chaperones, and transcription factors [281]. The ratio of GSH:GSSG in a resting cell is typically higher than 100:1; however, in different oxidative stress models, this ratio was shown to drop as low as 10:1, even to 1:1 [282].

### 5.5. Methionine Cycle and Transsulfuration Pathway

Methionine is an essential sulfur-containing amino acid that undergoes breakdown and regeneration through a series of metabolic reactions, known as the methionine cycle. In this process, methionine is first transformed into S-adenosyl-methionine (SAM), a universal methyl donor, which subsequently converts to S-adenosyl-homocysteine (SAH) after donating its methyl group. Hydrolysis of SAH then results in homocysteine (Hcy), which either undergoes a transsulfuration pathway to generate cysteine or, is converted back to methionine through a remethylation pathway with a methyl donation from the folate cycle [283]. Another enzyme that catalyzes the remethylation of Hcy to methionine is betaine-homocysteine-S-methyltransferase (BHMT) with betaine as the methyl donor. Additionally, the methionine salvage pathway or 5′-methylthioadenosine (MTA) cycle is involved in the regeneration of methionine from SAM as well as in the synthesis of polyamines [284,285].

The transsulfuration pathway, alternatively known as the cystathionine pathway, enables the cell to use methionine for the synthesis of GSH [286]. This pathway is particularly active in hepatocytes, and either absent or present in small quantities in other tissues outside the liver [287]. It represents a two-step process, converting Hcy into cysteine. In the first step, vitamin B6-dependent enzyme cystathionine β synthase (CBS) facilitates the synthesis of cystathionine by combining Hcy and serine. The second step involves the breakdown of cystathionine by a separate vitamin B6-dependent enzyme γ-cystathionase (also called cystathionine-γ-lyase, CGL), generating free cysteine for the synthesis of GSH [287].

### 5.6. Cysteine Metabolism and GSH Biosynthesis

The primary pathways by which cysteine is catabolized in the body include its utilization for GSH synthesis or its degradation through cysteine sulfinate-dependent pathways. Since the transsulfuration pathway converts nearly all methionine sulfur into cysteine sulfur before it is oxidized and eliminated, consuming a specific molar amount of methionine generates an almost equivalent molar amount of cysteine [288,289]. The enzyme cysteine dioxygenase (CDO) plays an important role in cysteine catabolism by oxidizing the sulfhydryl group of cysteine to cysteinesulfinate. This precursor is then used in the production of taurine and can be further transaminated into pyruvate and inorganic sulfur [290]. Taurine is an additional antioxidant that reduces oxidative stress by directly detoxifying ROS, enhancing GSH oxidation activity, and maintaining membrane permeability against ROS-induced disruptions [291]. The rate-limiting enzyme in GSH synthesis, glutamate-cysteine ligase (GCL), known as γ-glutamylcysteine synthetase (GCS), also utilizes cysteine as a substrate and hence competes with the enzyme CDO [292]. Thus, the activities of CDO and GCL are critical in regulating the partitioning of cysteine to meet diverse metabolic requirements and also to maintain its concentrations in the body [293]. These two enzymes, CDO and GCL, respond reciprocally with GCL activity decreasing and CDO activity increasing when the dietary protein or sulfur amino acid level is increased [294,295,296].

**Figure 10:**
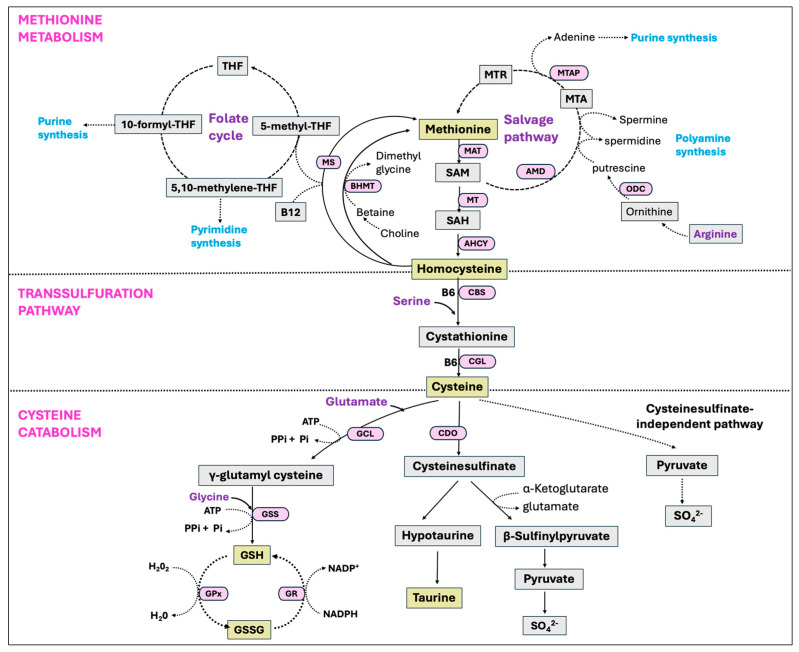
Methionine metabolism, transsulfuration pathway, and the cysteine catabolic pathways. Methionine is an essential amino acid that is converted to homocysteine through the transmethylation pathway. Homocysteine can then be reconverted back into methionine via remethylation pathways, involving a methyl donation from the folate cycle or the betaine. Methionine can also be regenerated via the salvage pathway involving the recycling of methylthioadenosine (MTA), a by-product of polyamine biosynthesis. Alternatively, homocysteine can be converted into cysteine via the transsulfuration pathway. Cysteine has multiple fates and is catabolized to produce various nonprotein compounds, such as glutathione (GSH), taurine, sulfate, and coenzyme A depending on the physiological and nutritional situations.

Glutathione (GSH), also known as γ-L-glutamyl-L-cysteinylglycine, is a tripeptide and the most abundant non-protein thiol that protects cells from oxidative injury, and is found in all mammalian tissues in the ranges of 1–10 mM with the highest concentrations in liver [297]. Synthesis of GSH occurs via a two-step ATP-dependent enzymatic reaction with the initial step driven by the rate-limiting enzyme GCL, catalyzing the formation of γ-glutamyl-L-cysteine from cysteine and glutamate. In the second step of GSH synthesis, the enzyme glutathione synthetase (GSS) catalyzes the addition of glycine to γ-glutamyl-L-cysteine, yielding GSH [297]. Despite its overexpression, GSS failed to elevate the GSH level, whereas the overexpression of GCL did result in a higher GSH level, indicating GCL as the rate-limiting enzyme in the process [298]. However, there is growing evidence that GSS is crucial in regulating the overall GSH synthesis in particular tissues or during stressful periods [299]. Glutathione is present as the thiol-reduced (GSH) and disulfide-oxidized (GSSG) forms, with the predominant thiol-reduced GSH accounting for nearly 98% of total GSH [300,301]. The antioxidant function of GSH is carried through GPx-catalyzed reactions, reducing peroxides with the oxidation of GSH to GSSG. The enzyme GR, in turn, restores GSH from GSSG by using NADPH as a reducing agent, therefore creating a continuous redox cycle [299]. All the major pathways, including the methionine cycle, transsulfuration pathway, cysteine catabolism, and GSH synthesis are summarized in Figure 10.

### 5.7. Findings in Chickens

Antioxidant mechanisms in chickens share fundamental similarities with those in mammals, as both rely on enzymatic and non-enzymatic antioxidant systems to combat oxidative stress [302]. A study demonstrated that pigeons have approximately double the activity of SOD and peroxidase compared to rats, while their CAT activity was considerably lower [303]. Early researchers had predicted that species with longer lifespans would possess a stronger antioxidant defense [304,305]. More recent studies between rats and pigeons, however, yielded inconsistent results. Instead of directly correlating with longevity, elevated antioxidant enzyme activity is now widely believed to be an adaptation to greater oxidative stress, suggesting the greater antioxidant defense as a possible indicator of increased oxidative stress rather than enhanced protection [306,307]. In a separate study, rats and pigeons showed similar plasma total antioxidant capacity (0.8 vs. 1.2 mM Trolox equivalents), but pigeons had higher antioxidant capacity (0.9 vs. 2.1 μmol Fe(II)/g tissue) in the skeletal muscle [308]. This study also showed that non-enzymatic antioxidants, including the plasma GSH, were higher in pigeons (11.8 vs. 19.9 mM), while enzymatic antioxidants such as SOD (94.1 vs. 16.8 U/g tissue) and GPx (34.7 vs. 4.6 U/g tissue) were generally higher in rats, especially in heart mitochondria. Moreover, rats showed significantly greater CAT levels in plasma (124.6 vs. 18.5 U/mL), heart (5469 vs. 1778 U/g tissue), and liver (218.6 vs. 34.1 U/g tissue) compared to pigeons. In contrast, pigeons had higher total skeletal muscle SOD (18.3 vs. 28.2 U/mg tissue) and heart SOD (136.8 vs. 271.5 U/g tissue). Despite such variations, total antioxidant status in plasma and liver were similar in both species due to counterbalancing contributions of non-enzymatic and enzymatic antioxidants [308].

Various studies have shown the elevated mRNA expression and enzymatic activity of SOD, CAT, and GPx2 in broilers reared under HS conditions [17,18,309,310]. There was a significantly higher enzymatic activity of SOD (1.30 vs. 1.72 U SOD/mg protein) and CAT (223.45 vs. 292.77 µmol H_2_O_2_/min/mg protein) without affecting the GPx activity (299 vs. 293 nmol NADPH oxidized/min × mg protein), while the GSH content (4.56 vs. 2.05 µg GSH/mg protein) was reduced in the liver of heat-stressed broilers [309]. However, a separate study found a decreased activity of GPx (9.62 vs. 5.6 U/mg protein) in the liver of heat-stressed chickens compared to control birds without significantly affecting the GR activity (0.27 vs. 0.17 U/mg protein) [311]. Higher *Trx* expression in the ovarian follicles of chicken has been linked with improved egg production rates [312]. However, the in vitro study revealed that chickens have extremely low TrxR activities compared to mammalian TrxR activity [313]. Moreover, the expression of *PrDx* is not tissue-specific in chickens, highlighting its crucial function as a ubiquitous housekeeping gene protective against oxidative injury [314]. In growing chickens, HS negatively affected the mitochondrial Trx system, including Trx2, TrxR2, and PrDx3 [315]. Acute HS was shown to upregulate the protein levels of PrDx1, PrDx3, and PrDx4 in the small yellow follicles of layer chickens [316], whereas chronic HS significantly downregulated the relative mRNA expression of *NADPH* and *GPx* genes in broiler chickens [17]. Further, separate studies found the enzymatic activity of GR, GSH, GSSG, and GSH:GSSG ratio to be adversely affected during HS [14,245].

The first limiting amino acid in a typical corn–soya-based poultry feed is methionine, which participates in an important biosynthesis pathway of other essential molecules. Multiple studies have reported the dose-dependent beneficial effects of different isoforms of methionine supplementation in poultry diets during the time of oxidative stress [317,318,319]. Supplementing broilers under acute HS conditions with adequate and high levels of DL-methionine increased the expression of *GSS* and *GPx-7* genes [320]. Cysteine can be produced endogenously from the transsulfuration pathway [321,322]; therefore, it is not considered an essential amino acid in poultry feed. However, for GSH synthesis, the availability of cysteine is regarded as the rate-limiting factor among the three amino acids-cysteine, glutamate, and glycine [323,324]. During periods of oxidative stress, the need for GSH is greater, and cysteine may be limiting when attempting to produce adequate levels of GSH. Among many amino acids, cysteine showed the highest incorporation into the tissues of chickens during HS [325]. Furthermore, there was an increased expression of the hepatic *CBS* gene in chickens in response to a cysteine-deficient diet [326], suggesting the active intracellular compensatory mechanism to produce sufficient levels of cysteine through the transsulfuration pathway. Numerous studies have identified the beneficial effects of different isoforms of cysteine, such as N-acetylcysteine [327,328] and S-allyl-cysteine [329], under HS conditions. Arginine is also a limiting amino acid in poultry diet because birds lack a functional urea cycle, and are thus unable to synthesize endogenous arginine [330]. Arginine was shown to increase the antioxidant potential of birds by slowing down the cellular nitric oxide breakdown [331], and by increasing the levels of GSH [332]. Various amino acids, phytochemicals, and natural compounds that enhance antioxidant defense mechanisms when supplemented to chickens are summarized in Table 1.

## 6. The Antioxidant Network and Energy Metabolism

The principal roles of antioxidants include the prevention of ROS production, free radicals scavenging before they react with biological molecules, and repairing the damage. To achieve these functions, different antioxidants work together within the defense network cooperatively and synergistically. The interaction between different antioxidants helps them to work efficiently against oxidative stress, while also restoring their original properties. This process is generally referred to as the antioxidant network [345,346]. Vitamin E (α-tocopherol) works as a lipophilic chain-breaking antioxidant and prevents peroxidative damage to cell membranes by neutralizing free radicals via hydrogen atom transfer [347]. In doing this, vitamin E produces a nonradical molecule and an α-tocopherol radical, which can interact with another radical to form stable compounds, attack a lipid molecule, or regenerate vitamin E by interacting with reducing agents like vitamin C or ubiquinol [348]. Reports suggest that vitamin E and vitamin C operate together cyclically to achieve the antioxidant function [349,350,351]. Additionally, this interaction between vitamin E and vitamin C is important to recycle and replenish the antioxidant potential of vitamin E.

Vitamin C (ascorbate/ascorbic acid) is a water-soluble antioxidant that contains radical scavenging activity. In addition to directly deactivating the peroxyl radicals formed in the aqueous phase, ascorbate can also recycle α-tocopherol from its radical form (α-tocopherol radical) [352,353]. Ascorbate, upon donating an electron to α-tocopherol, becomes oxidized to a less reactive compound called semidehydroascorbate (SDA) radical. This SDA radical has two potential routes; it can either be regenerated back to ascorbic acid or converted to dehydroascorbate (DHAA). The enzyme DHAA reductase can then catalyze the regeneration of ascorbic acid from DHAA by utilizing GSH as a reducing agent [354,355,356]. The series of oxidation–reduction reactions enabling ascorbic acid and GSH to interact with each other has been well elucidated [357,358,359]. In addition, NADPH, mainly derived from the PPP, plays a key role in the antioxidant network by providing reducing equivalents to restore the reduced forms of GSH or Trx. This series of interconnected reactions regenerates the reduced compounds, thereby enhancing their antioxidant properties (Figure 7). Oxidative stress has been shown to deplete both the NADPH pool and the NADPH:NADP^+^ ratio, while also altering NADP-dependent metabolic pathways [360,361,362]. Moreover, to regenerate NADPH during the oxidative stress, there was a shift in glycolytic flux toward the oxidative PPP [362,363,364].

### Findings in Chickens

Birds can endogenously synthesize vitamin C, although its level depletes quickly during the HS condition [365]. In contrast, chickens cannot synthesize vitamin E, rather they rely on dietary intake to fulfill their requirements [366,367]. Numerous studies revealed the protective role of vitamin E and vitamin C during the period of oxidative stress in chickens [368,369,370]. Vitamin E has been reported to enhance growth rates, egg production, and hatchability [371,372,373]. Dietary supplementation of vitamin E in poultry feed increased α-tocopherol levels, while the MDA concentration was decreased in tissues and serum [373,374,375]. In another study, vitamin E has been found to reduce oxidative stress and histopathological alterations in the duodenum and jejunum induced by the Newcastle disease virus [376]. Vitamin C, on the other hand, has been shown to lower high plasma corticosterone levels, and to maintain normal leukocytic count in chickens [377,378]. The combined supplementation of vitamin C (200 mg/kg) and vitamin E (250 mg/kg) was more effective than individual supplementation in enhancing erythrocytic antioxidant status in colored broiler breeders subjected to hot and humid conditions [370]. Moreover, as compared to supplementation with vitamin E (125 IU/Kg) alone, a combination of vitamin C (200 mg/kg) and vitamin E (125 IU/kg) synergistically reduced the activity of lipid peroxidase (1.7 vs 1.54 nmol/MDA/mg protein) while increasing GR activity (71.08 vs. 79.82 U/mL) in layers under tropical summer conditions [379]. Enzymes and metabolites associated with energy metabolism, such as glucose, glucose-6-phosphate (G6P), and the member of the pentose phosphate pathway were found to be elevated during HS in chickens [248,380]. Moreover, a separate study demonstrated that glucose supplementation during chronic HS improved the production parameters possibly by shifting the excess glucose into protein synthesis [23].

## 7. Ubiquitin–Proteasome System (UPS) and the Molecular Chaperones

Protein oxidation from free radicals typically results in structural damage and functional alterations, thereby affecting protein homeostasis (proteostasis). This disturbance can lead to numerous detrimental effects on cells, including the functional loss of essential proteins or the toxic gain-of-function caused by misfolded protein conformations [381,382]. However, cells have protective mechanisms, known as protein quality control systems, which can prevent proteins from misfolding and toxic aggregations induced by stressful stimuli, including oxidative stress. This quality control system depends on three main strategies: the proteolytic system-mediated degradation of misfolded proteins, the ATP-dependent chaperone-mediated refolding of non-native proteins, and their sequestration into inert inclusions [383,384]. One of the primary pathways in the clearance of misfolded proteins is the ubiquitin–proteasome system (UPS), which depends on the ubiquitination of misfolded proteins followed by 26S proteasomal degradation [385]. Studies showed that the degradation of misfolded protein through the proteasome accelerated over ten-fold upon exposure to H_2_O_2_ or O_2_^•−^ [386].

Molecular chaperones are proteins that assist with the folding and/or assembling of other macromolecules into more orderly structures, without themselves being a part of these final structures [387]. Chaperons can be broadly divided into two groups based on their dependency on metabolic energy: those depending on the metabolic energy, e.g., all ATP-dependent chaperones, and those independent of metabolic energy, e.g., small heat shock proteins (sHSPs) and protein disulfide isomerase [381,388,389]. ATP-dependent chaperones can either be constitutively expressed to perform the essential housekeeping roles or induced by brief exposure to increased temperatures or other stressors that lead to protein denaturation. Chaperones that are expressed constitutively are termed heat shock cognates (HSCs), whereas stress-inducible chaperones are referred to as heat shock proteins (HSPs) [390].

**Figure 11:**
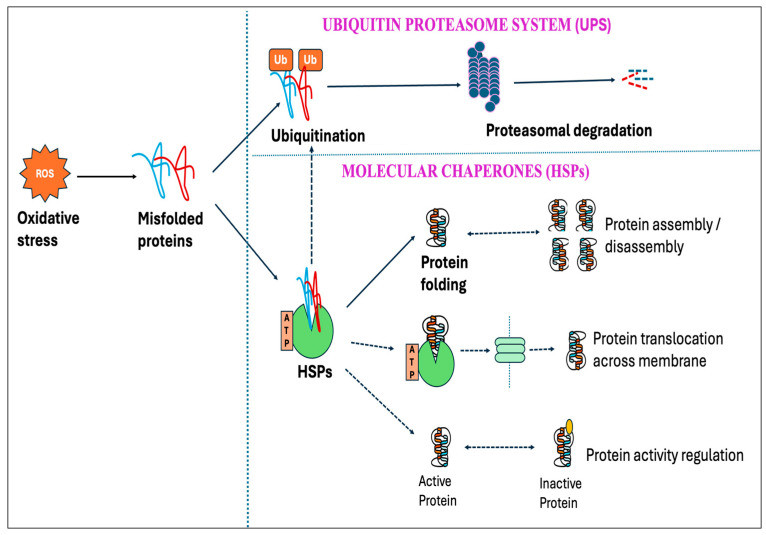
Schematic representation of the ubiquitin–proteasome system (UPS) and the molecular chaperones in regulating proteostasis. Misfolded protein resulting from oxidative injury activates the UPS, leading to the ubiquitination of misfolded proteins and 26S proteasomal degradation. The primary role of molecular chaperones, including the heat shock protein 70 (HSP70) and heat shock protein 90 (HSP90), is to refold the misfolded proteins; however, they have also been demonstrated to assist in the degradation of misfolded proteins through the UPS. In addition, HSPs play roles in protein assembly/disassembly, their translocation across membranes, and activity regulation of secretory proteins.

**Figure 12:**
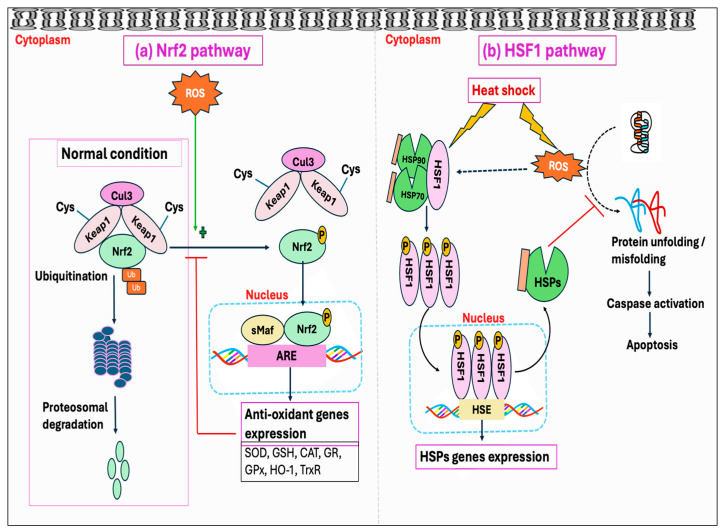
Schematic representation of the nuclear factor erythroid 2-related factor 2 (Nrf2) pathway and heat shock factor 1 (HSF1) pathway in regulating oxidative stress and proteotoxic stress, respectively: (**a**) Under normal conditions, kelch-like ECH-associated protein 1 (Keap1) binds with Nrf2, which will be subsequently ubiquitinated by Cullin 3 (Cul3) ligase for proteasomal degradation. During oxidative stress, reactive oxygen species (ROS) modifies cysteine residues in Keap1, leading to Nrf2 stabilization, nuclear translocation, and target gene binding to trigger the expression of multiple antioxidant genes. (**b**) HSF, particularly HSF1, is activated by both proteotoxic stimuli such as heat shock and oxidative stress. Upon activation, it trimerizes and interacts with the heat shock element (HSE) of its target genes in the nucleus, thereby regulating the expression of heat shock proteins (*HSPs*). As a molecular chaperone, HSPs assist in proper protein folding and mitigate proteotoxic stress.

Among different HSPs, HSP70 and HSP90 are the most important chaperones, which are highly conserved and ubiquitous in all organisms for the maintenance of life [391,392]. These chaperones work cooperatively and execute a wide range of functions, such as folding and assembly of newly made proteins, refolding of misfolded or aggregated proteins, facilitating the translocation of organelle and secretory proteins across membranes, and regulating specific protein activities (Figure 11) [393,394,395,396,397]. Although the major function of HSPs is to help improperly folded proteins recover their normal folded structure, they also cooperate with the UPS system to enhance the removal of terminally misfolded proteins [398,399]. Besides their importance in protein folding, HSP70 and HSP90 also function as a negative regulator for heat stress transcription factors (HSFs) by maintaining the inactive state of HSFs (Figure 12) [400,401]. Furthermore, experimental findings have indicated an association between HSP70 and redox status, where the HSP70 significantly enhanced the enzymatic activities of GPx and GR [402].

### Findings in Chickens

The skeletal muscle of long-lived pigeons demonstrated significantly lower proteasome activity than short-lived rats, suggesting the higher protein oxidation levels in pigeons result from reduced proteasome activity rather than reduced ROS production [403]. However, the ubiquitin and *HSP70* expression increased upon heat shock in chicken testicular cells but not in mouse testicular cells, contributing to greater thermotolerance in avian spermatogenesis [404]. While there are numerous studies demonstrating the role and expression of *HSPs*, there is limited information on the role of the UPS system in degrading the misfolded protein during HS conditions in chickens. One of the studies showed that mitochondrial O_2_^•−^ production promotes the expression of *atrogin-1* mRNA in HS-cultured muscle cells, leading to protein degradation via the UPS pathway [405]. Moreover, cortisol-induced mitochondrial O_2_^•−^ production also resulted in UPS activation and subsequent degradation of muscle proteins in broiler chickens [406]. Moreover, an increased level of HSP70 was found to protect the intestinal mucosa from HS damage by enhancing the antioxidant enzyme activity and inhibiting the progression of lipid peroxidation [407]. Researchers also examined *HSP70* expression in avian erythroid cells during their maturation, demonstrating that definite red blood cells react to heat shock by increasing the synthesis of HSP70 protein ten-to-twenty-fold without significantly changing the *HSP70* mRNA levels [408]. However, a separate study found that *HSP70* members were among the most downregulated genes within the ileal transcriptome of HS birds compared to the control group [409]. Multiple studies have agreed that the long-term upregulation of *HSP70* is associated with improved thermotolerance acquisition in chickens [410,411,412].

## 8. Transcriptional Regulation of Oxidant and Antioxidant Mechanisms

Several transcription factors are involved in regulating the oxidant and antioxidant mechanisms during oxidative stress. The major transcription factors/pathways include nuclear factor erythroid 2-related factor 2 (Nrf2), nuclear factor kappa B (NF-κB), activator protein-1 (AP-1), mitogen-activated protein kinase (MAPK), and forkhead box O (FoxO) [413,414,415]. Moreover, a family of heat shock factors (HSFs) drives proteotoxic stress-inducible transcription [416]. Once activated, these transcription factors induce the expression of multiple genes related to redox homeostasis, inflammation, immunity, cell survival, and apoptosis [413].

### 8.1. Nuclear Factor Erythroid 2-Related Factor 2 (Nrf2) Pathway

The Nrf2 is widely recognized as the redox-sensitive master regulator of cellular responses against oxidative stress [417]. During normal physiological conditions, kelch-like ECH-associated protein 1 (Keap1) attaches to Nrf2 in the cytoplasm and prevents it from nuclear translocation, leading to the ubiquitination and proteasomal degradation of Nrf2 [418,419]. During oxidative stress, the modification of specific stress-sensing cysteine residues results in conformational changes in Keap1 [420,421], thereby inhibiting Keap1 from facilitating the Nrf2 ubiquitination by Cullin 3 (Cul3) [422]. As a result, Nrf2 stabilizes and translocates into the nucleus where it heterodimerizes with small Maf proteins (sMaf) and binds to the antioxidant response element (ARE) within the promoter region of its target gene, resulting in the rapid activation of antioxidant genes, including *SOD*, *CAT*, *GPx*, and *GSH*, associated with ROS detoxification (Figure 12a) [311,423].

### 8.2. Heat Shock Factors (HSFs) Pathway

Proteotoxic stress disrupts proper protein folding, leading the misfolded proteins to accumulate and aggregate. Heat shock factors (HSFs) are the master trans-activators that are triggered by multiple proteotoxic stimuli, including heat shock [424]. In response, HSFs quickly activate the transcription of genes that encode HSPs, which subsequently operate as molecular chaperones [416]. In mammalian systems, three HSFs, namely, HSF1, HSF2, and HSF4, have been identified [425]; however, chickens have an additional HSF3, which is co-expressed with HSF1 in chicken cells and tissues [426]. This suggests that both *HSF1* and *HSF3* are the primary genes of the HSF family in chickens involved in heat shock response [426,427]. Among these various HSFs, HSF1 is considered the principal regulator of HSP expression, while HSF2 heterodimerizes with HSF1 and regulates specific *HSP* gene expression [428,429]. Moreover, HSF1 is also activated in response to oxidative stress besides heat shock response and other proteotoxic stresses [430]. These stressful stimuli trigger HSF1 to dissociate from HSP90 and HSP70, thereby activating HSF1. HSF1 then trimerizes, followed by several post-translational modifications and binding to the heat shock elements (HSEs) in the promoter region of its target genes (Figure 12b). This results in the transcriptional activation of target genes by HSF1 [416,431,432].

### 8.3. Nuclear Factor Kappa B (NF-κB) Pathway

**Figure 13:**
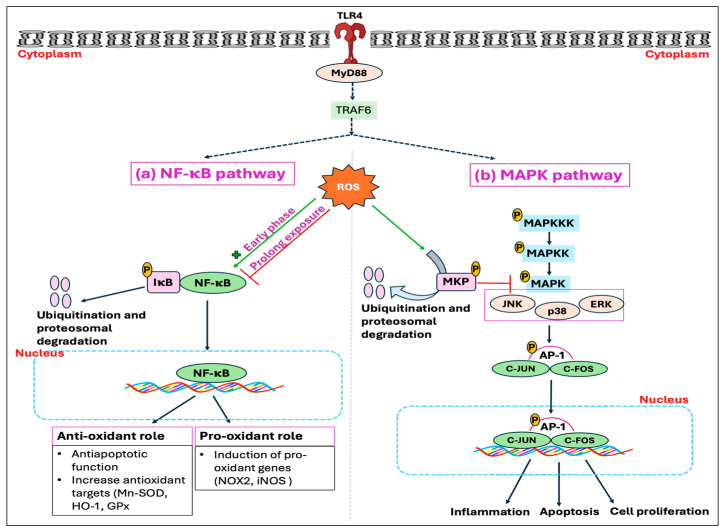
Schematic representation of the nuclear factor kappa B (NF-κB) pathway and mitogen-activated protein kinase (MAPK) pathway in regulating oxidative stress: (**a**) Reactive oxygen species (ROS)-induced oxidative stress has a dual role in NF-κB activity; the early phase activates the NF-κB pathway while prolonged exposure can inhibit the degradation of the inhibitor of nuclear factor kappa B (IκB), thus preventing NF-κB activation. Additionally, the NF-κB pathway can exert both antioxidant as well as pro-oxidant functions during the time of oxidative stress by regulating the expression of various antioxidant genes and pro-oxidant genes, respectively. (**b**) Activation of another transcription factor, activator protein-1 (AP-1), is linked with cellular proliferation, inflammation, and death. AP-1 is the primary target of MAPK, which has three subfamilies, namely, extracellular signal-regulated kinase (ERK), c-Jun N-terminal kinase (JNK), and p38. One mechanism by which oxidative stress triggers MAPK and AP-1 is the inactivation and degradation of mitogen-activated protein kinase phosphatase (MKP). Under normal conditions, MKP negatively regulates MAPK by dephosphorylation, thereby rendering its activity. However, ROS can promote the degradation of MKP, resulting in the activation of MAPK signaling.

The NF-κB proteins represent a group of transcription factors that have a key role in regulating both inflammation and immunity. Generally, the activity of NF-κB is regulated through IκB proteins, which typically prevents NF-κB from its DNA binding. However, the activation of NF-κB through a canonical pathway causes IκB proteins to phosphorylate, ubiquitinate, and degrade subsequently in the proteasome. Consequently, activated NF-κB moves into the nucleus and triggers the activation of target genes [433,434]. Free radicals have a dual role in NF-κB activity, either stimulating or suppressing it in a phase- and context-dependent manner. Particularly, the initial phase of oxidative stress triggers the activation of the NF-κB pathway while prolonged oxidative stress could inhibit proteasomal activity, thereby blocking the degradation of IκB and consequently preventing NF-κB activation [435]. Moreover, the NF-κB pathway has the potential to exhibit both antioxidant and pro-oxidant roles during the time of oxidative stress [436]. Antioxidant roles of NF-κB include anti-apoptotic function via downregulation of the JNK pathway and reduction in ROS accumulation via antioxidant targets, including the *Mn-SOD*, heme oxygenase-1 (*HO-1*), and *GPx* [437,438]. Conversely, NF-κB signaling may exert a pro-oxidant role by activating the expression of certain genes like *NOX2*, *XO*, and *iNOS* (Figure 13a) [439,440,441].

### 8.4. Mitogen-Activated Protein Kinase (MAPK) Pathway

The ubiquitous protein complex AP-1 is activated by numerous signals and mediates various cellular responses. It comprises a group of related dimeric complexes from the FOS and JUN protein families rather than a singular transcription factor [442]. Enhanced activity of AP-1 has been linked to cellular proliferation, differentiation, stress, and death [443,444]. Various growth factors and proinflammatory cytokines, as well as UV radiation, have been shown to activate AP-1 activity through a range of distinct mechanisms. This AP-1 represents one of the major targets of the mitogen-activated protein kinases (MAPKs), a group of serine/threonine protein kinases that are closely engaged in regulating various cellular processes [445]. Three main subfamilies of MAPK, namely, extracellular signal-regulated kinase (ERK), c-Jun N-terminal kinase (JNK), and p38, are identified. The activation of each MAPK subgroup occurs through a series of phosphorylation steps, starting from the activation of MAPK kinase kinases (MAPKKK). Therefore, the dephosphorylation of MAPKs by phosphatases could be the most effective mechanism for its negative regulation. Evidence from studies suggested that ROS can trigger or facilitate the induction of MAPK signaling pathways [446,447]. One of the potential mechanisms for ROS-mediated MAPK activation involves inactivation and degradation of the mitogen-activated protein kinase phosphatase (MKP), resulting in the long-term activation of the JNK, p38, and ERK pathways (Figure 13b) [448,449].

### 8.5. Findings in Chickens

The protective effects of different polyphenols and carotenoids in poultry have been shown to mediate through the Nrf2 pathway. Elevating dietary lycopene levels mitigated the adverse effects of HS on the Nrf2 system, and had a positive impact on chicken performance [337,450]. Moreover, dietary resveratrol has been shown to alleviate heat-induced liver injury through the Nrf2–Keap1 pathway [336]. On the other hand, cadmium, an environmental pollutant, induced oxidative stress and led to the upregulation of *Nrf2* and downstream target genes in the chicken’s kidney [451]. However, *Nrf2* showed a slight downregulation on day 1 post-HS, while no significant difference was observed in the mRNA expression of *Nrf2* at 12 days post-HS in broiler chickens [17]. Under HS conditions, these genes were over-expressed in backyard chicken breeds that proved to be more resistant to high temperatures compared with commercial broilers [412]. Furthermore, thermal manipulation with cold stress in broiler chickens decreased the hepatic *HSF3* expression, while the splenic *HSF3* remained unaffected [452].

The expression of chicken myeloid differentiation factor 88 (*MyD88-2*) was observed in various tissues, and the overexpression of *MyD88-2* was shown to activate NF-κB significantly [453]. Both the mRNA expressions and protein levels of NF-κB and other inflammatory genes were significantly higher in the hypothalamus of the HS group than in the control group [454]. Similarly, at 35 and 42 days of age, the HS group exhibited a substantial increase in the relative expression of *MyD88* and *NF-κB* genes compared to the control group [455]. A separate study demonstrated increased mRNA levels of toll-like receptor 4 (*TLR*4), *C-JUN*, *C-FOS*, *caspase*-3, and *p38* of MAPK in the jejunal mucosa of black-boned chicken under HS condition [456]. Moreover, the mRNA expressions of NF-κB subunits (*p50*, *p65*, *p52*, and *RelB*), AP-1 (*C-JUN* and *C-FOS*), and *IRF3* were significantly elevated in the spleen of chickens under HS when compared with the control group [457].

## 9. Conclusions

Heat stress is becoming a major threat for both humans and animals. In livestock production, HS is directly related to higher mortality rates and decreased production, imposing not only economic burdens but also increasing food insecurity on a global scale. Mitigating the adverse effects of HS requires an in-depth understanding of major pathways related to ROS production and detoxification. Heat stress triggers various cellular enzymatic systems, leading to the excessive production of ROS and subsequent oxidative injury to lipids, proteins, carbohydrates, and DNA. However, the exact mechanisms of how HS stimulates the enzymatic systems to generate excessive amounts of ROS, ultimately developing into oxidative stress, have not been fully uncovered. Animals, including chickens, fight against HS through different physiological and hormonal responses, such as panting, increased peripheral circulation, reduced feed intake, and secretion of stress hormones. However, these adaptive responses have been directly or indirectly linked to the development of oxidative stress. A detailed investigation into these initial responses against HS and an exploration of their downstream mechanisms may establish the connection between HS and oxidative stress in chickens. Conversely, cells also have enzymatic and non-enzymatic antioxidant systems that detoxify ROS/RNS, thereby protecting cells from oxidative injury. The interconnected series of reactions between various antioxidants, such as the antioxidant network, enhance the potency of each antioxidant in combating oxidative stress. The process of ROS/RNS generation and elimination is tightly regulated through multiple transcriptional factors, and there exists a significant interplay between different systems in maintaining the redox homeostasis of cells. Extensive studies in chickens have reported the mRNA expression and protein activity of different oxidant and antioxidant genes. Although the primary mechanisms of oxidative stress and antioxidant defense mechanisms between chickens and mammals have fundamental similarities, further molecular studies are required to identify any unique pathways in chickens and to develop effective nutritional interventions against HS.

## Figures and Tables

**Table 1:** Overview of nutritional supplements that enhance antioxidant capacity in chickens under heat stress conditions.

Supplements	Experimental Model	Antioxidant Effects	Reference
***Amino acids***			
DL-Methionine (DL-Met)	Broilers (Cobb 500) HS (38 °C for 24 h) Diets (starter diet: 0%, 2.95%, and 10% DL-Met; grower diet: 0%, 2.75%, and 10% DL-Met)	Both the normal (2.95% to 2.75%) and excess (10%) DL-Met diets increased the expression of *CBS*, *GSS*, and *GPx7* genes in heat-stressed birds	[320]
In ovo methionine-cysteine injection	Broilers (Ross) HS (39.6 °C for 6 h daily between 10 and 18 days of incubation) Injection (no, 0.75% saline, and 5.9 mg L-methionine + 3.4 mg L-cysteine at day 17.5 of incubation)	Met-Cys increased the expression of *GPx*, *SOD*, and GSH:GSSG ratio in serum and tissues while reducing MDA levels in tissues	[333]
N-acetyl-l-cysteine (NAC)	Layers (Hy-Line Brown) HS (36 °C from 8 a.m. to 6 p.m. every day for three weeks) Diets (0 and 1 g/kg NAC)	NAC diet upregulated the activity of Nrf-2, SOD2, GPx, CAT, and TAC while decreasing the MDA contents and the expression levels of *HSP70* and proinflammatory cytokines like *IL-8*, *IL-18*, and *TNF-α* in the ovaries	[327]
Arginine	Ganders (Geese) HS (37.7 °C for two months) Diets (0, 0.2, and 0.4 g/kg arginine)	Plasma TAC capacity and SOD and CAT activities were increased, whereas MDA and cortisol levels were decreased by increasing concentrations of arginine	[334]
Glutamine	Broilers (Arbor Acres) HS (34 °C for 24 h) Diets (0, 5, 10, and 20 g/kg glutamine)	20 g/kg glutamine reduced MDA levels while increasing TAC, GSH, GPx, and GS levels in the skeletal muscles of heat-stressed birds, compared to 0 g/kg glutamine	[335]
***Phytochemicals***			
Resveratrol	Broilers HS (35 °C, 8 h/day for 7 days) Diets (0 and 400 mg/kg resveratrol)	Resveratrol diet increased hepatic Nrf2 and HO-1 levels, enhanced GPx and SOD activities, and reduced MDA levels. Additionally, it lowered protein levels of Keap1, HSP70, and p62	[336]
Lycopene	Broilers (Ross 308) HS (34 °C for 8 h a day for 42 days) Diets (0, 200, and 400 mg/kg lycopene)	Increased activities of muscle SOD and GPx while reducing MDA concentration in a dose-dependent manner. Further, it increased muscle *Nrf2* expression and reduced *Keap1* expression	[337]
Genistein	Japanese quail HS (34 °C for 8 h a day for 32 days) Diets (0, 200, 400 and 800 mg/kg genistein)	Genistein decreased serum and hepatic MDA levels while increasing the levels of serum vitamins C, E, and A as compared to controls	[338]
*Schisandra chinensis* (SC) and *Ligustrum lucidum* (LL)	Layers (Hy-Line) HS (32 °C for 28 days) Diets (basal, 1% SC and 1% LL)	SC or LC diets lowered MDA levels in serum, tissues, and egg yolk while increasing the GR activity in serum and tissues of heat-stressed birds compared to basal diet	[339]
Astaxanthin (AST)	Broilers (Ross 308) HS (32.8 °C for 8 h a day at week 4 and 30.2 °C for 8 h a day at week 5) Diets (0, 20, 40, and 80 ppm AST)	Increasing levels of AST led to a linear reduction in plasma and muscle MDA levels while increasing the plasma CAT and SOD levels. In muscle, it increased TAC without affecting the levels of other antioxidants	[340]
Curcumin	Broilers (Arbor Acres) HS (34 °C for 8 h a day for 20 days) Diets (0, 50, 100, and 200 mg/kg curcumin)	Curcumin diets increased the levels of hepatic GSH, γ-GCL, GPx, and GST while reducing the levels of serum MDA in heat-stressed birds. Moreover, they upregulated the expressions of *Nrf2*, *CAT*, and *HO-1* genes in liver	[311]
Dihydroquercetin (DHQ)	Broilers (Ross 308) HS (continuous 35 °C for 21 days) Diets (basal, 0.5 g/kg DHQ substrate, 0.3 g/kg vitamin E and 0.5 g/kg DHQ substrate + 0.3 g/kg vitamin E)	DHQ supplementation increased blood GPx level by 13% and TAC by 33% in heat-stressed chickens compared to no DHQ diet	[341]
***Other compounds***			
Taurine	Broiler (Arbor Acres) HS (34 °C for four weeks) Water (0, 0.5, 2, and 8 g/L taurine)	2 g/L taurine increased TAC, GPx, CAT, and SOD levels compared to 0 g/L supplementation	[342]
Betaine	Dual-purpose roosters (Mandarah) HS (38 °C for 4 h a day, 3 consecutive days each week, over a period of 20 weeks) Diets (basal, 1000 mg/kg betaine, betaine + vit C, betaine + vit E, and betaine + vit C + vit E)	Betaine-containing diets (with or without vitamins) increased seminal plasma TAC while recovering the blood plasma MDA levels compared to HS birds with basal diet	[343]
Selenium	Quails HS (38 °C for 24 h) Diets (0.11 and 0.33 mg/kg selenium)	Compared to selenium deficient diet (0.11 mg/kg), selenium sufficient diet (0.33 mg/kg) increased the hepatic expression of *GSS*, *GR*, and *UCP* genes in heat-stressed quails	[344]

## Abbreviations

The following abbreviations are used in this manuscript:

ADP	Adenosine diphosphate
AGEs	Advanced glycation end products
AHCY	Adenosylhomocysteinase
AMD	Adenosylmethionine decarboxylase
AP-1	Activator protein-1
ARE	Antioxidant response element
ATF6	Activating transcription factor 6
ATP	Adenosine triphosphate
BCL2	B-cell lymphoma 2
BH4	Tetrahydrobiopterin
BHMT	Betaine-homocysteine methyltransferase
CAT	Catalase
CAT-1	Cationic amino acid transporter 1
CBS	Cystathionine-beta-synthase
CDO	Cysteine dioxygenase
C-FOS	c-Fos proto-oncogene
CGL	Cystathionine-gamma-lyase
C-JUN	c-Jun proto-oncogene
CO_3_^•−^	Carbonate radical
CoQ	Ubiquinone
CoQH_2_	Reduced coenzyme Q (ubiquinol)
COX	Cyclooxygenase
Cul3	Cullin 3
Cyt.C	Cytochrome C
DHAA	Dehydroascorbate
DNA	Deoxyribonucleic acid
eNOS	Endothelial nitric oxide synthase
ER	Endoplasmic reticulum
ERK	Extracellular signal-regulated kinase
ETC	Electron transport chain
FADH2	Flavin adenine dinucleotide
FFAs	Free fatty acids
FKBP	FK506 binding proteins
FoxO	Forkhead box O
GC	Glucocorticoids
GCH1	GTP cyclohydrolase I
GCL	Glutamine cysteine ligase
GPx	Glutathione peroxidase
GR	Glutathione reductase
GREs	Glucocorticoid response elements
Grx	Glutaredoxin
GSH	Reduced glutathione
GS	Glutamine synthetase
GSS	Glutathione synthetase
GSSG	Oxidized glutathione
GST	Glutathione S-transferase
H^+^	Hydrogen ion
HCO_3_^−^	Bicarbonate ion
HO^•^	Hydroxyl radical
H_2_O_2_	Hydrogen peroxide
HS	Heat stress
HSC	Heat shock cognate
HSE	Heat shock element
HSF	Heat shock factor
HSP	Heat shock protein
IκB	Inhibitor of nuclear factor kappa B
IL-8	Interleukin-8
IL-18	Interleukin-18
iNOS	Inducible nitric oxide synthase
IP_3_R	Inositol 1,4,5-trisphosphate receptors
IRE1	Inositol requiring 1 protein
JNK	c-Jun N-terminal kinase
Keap1	Kelch-like ECH-associated protein 1
LOO^•^	Lipid peroxyl radical
LOX	Lipoxygenase
MAMs	Mitochondria-associated ER membranes
MAPK	Mitogen-activated protein kinase
MAPKK	MAPK kinase
MAPKKK	MAPK kinase kinase
MAT	Methionine adenosyl transferase
MDA	Malondialdehyde
MKP	Mitogen-activated protein kinase phosphatase
mPTP	Mitochondrial permeability transition pore
MT	Methyl transferase
MTAP	Methylthioadenosine phosphorylase
MTR	Methylthioribose
NADH	Nicotinamide-adenine dinucleotide
NADPH	Nicotinamide adenine dinucleotide phosphate
NF-κB	Nuclear factor kappa-light-chain-enhancer of activated B cells
^•^NO	Nitric oxide radical
^•^NO_2_	Nitrogen dioxide radical
NOS	Nitric oxide synthase
nNOS	Neuronal nitric oxide synthase
NOX	NADPH oxidase
Nrf2	Nuclear factor erythroid 2-related factor 2
O_2_^•−^	Superoxide radical
ODC	Ornithine decarboxylase
8-OHdG	8-hydroxy-2′-deoxyguanosine
ONOO^−^	Peroxynitrite
p62	Sequestosome-1
PC	Protein carbonyl
PDI	Protein disulfide isomerase
PERK	PKR-like ER kinase
PI_3_K	Phosphatidylinositol 3-kinase
PPP	Pentose phosphate pathway
PrDx	Peroxiredoxin
ROO^•^	Peroxyl radical
RNS	Reactive nitrogen species
ROS	Reactive oxygen species
RyR	Ryanodine receptors
SAH	S-adenosyl-homocysteine
SAM	S-adenosyl-methionine
SDA	Semidehydroascorbate
SIRT3	Sirtuin 3
sMaf	small Maf protein
SOD	Superoxide dismutase
TAC	Total antioxidant capacity
THF	Tetra-hydrofolate
TNF-*α*	Tumor necrosis factor-alpha
Trx	Thioredoxin
TrxR	Thioredoxin reductase
Ub	Ubiquitin
UCP	Uncoupling proteins
UPR	Unfolded protein response
UPS	Ubiquitin–proteasome system
XO	Xanthine oxidase
